# Defining the functional properties of cyclopropane fatty acid synthase from *Pseudomonas aeruginosa* PAO1

**DOI:** 10.1016/j.jbc.2024.107618

**Published:** 2024-07-31

**Authors:** Vivian Ezeduru, Annie R.Q. Shao, Felipe A. Venegas, Geoffrey McKay, Jacquelyn Rich, Dao Nguyen, Christopher J. Thibodeaux

**Affiliations:** 1 Department of Chemistry, McGill University, Montreal, Quebec, Canada; 2 Research Institute of the McGill University Health Center, McGill University, Montreal, Quebec, Canada; 3 Department of Microbiology and Immunology, McGill University, Montreal, Quebec, Canada; 4 Department of Medicine, McGill University, Montreal, Quebec, Canada; 5 Centre de Recherche en Biologie Structurale, McGill University, Montreal, Quebec, Canada

**Keywords:** *Pseudomonas aeruginosa*, enzyme catalysis, lipid metabolism, membrane, structural biology, bioinformatics, bacterial metabolism, structure-function, mass spectrometry, infectious disease

## Abstract

Cyclopropane fatty acid synthases (CFAS) catalyze the conversion of unsaturated fatty acids to cyclopropane fatty acids (CFAs) within bacterial membranes. This modification alters the biophysical properties of membranes and has been correlated with virulence in several human pathogens. Despite the central role played by CFAS enzymes in regulating bacterial stress responses, the mechanistic properties of the CFAS enzyme family and the consequences of CFA biosynthesis remain largely uncharacterized in most bacteria. We report the first characterization of the CFAS enzyme from *Pseudomonas aeruginosa* (PA), an opportunistic human pathogen with complex membrane biology that is frequently associated with antimicrobial resistance and high tolerance to various external stressors. We demonstrate that CFAs are produced by a single enzyme in PA and that *cfas* gene expression is upregulated during the transition to stationary phase and in response to oxidative stress. Analysis of PA lipid extracts reveal a massive increase in CFA production as PA cells enter stationary phase and help define the optimal membrane composition for *in vitro* assays. The purified PA-CFAS enzyme forms a stable homodimer and preferentially modifies phosphatidylglycerol lipid substrates and membranes with a higher content of unsaturated acyl chains. Bioinformatic analysis across bacterial phyla shows highly divergent amino acid sequences within the lipid-binding domain of CFAS enzymes, perhaps suggesting distinct membrane-binding properties among different orthologs. This work lays an important foundation for further characterization of CFAS in *P. aeruginosa* and for examining the functional differences between CFAS enzymes from different bacteria.

Cyclopropane fatty acids (CFAs) (1, 2) are widely distributed membrane components in bacteria, where they protect against low pH, extreme temperatures, and a variety of chemical and environmental perturbations (3, 4, 5). CFAs are also present in the seed oils of certain plants (5) and in several species of *Leishmania* (6), where they contribute to intracellular survival of the parasite. CFAs are thought to exert these protective effects by altering the chemical stability and biophysical properties of bacterial membranes (7, 8, 9), primarily by reducing membrane permeability through the ordering of acyl chains in lipid bilayers without significantly altering membrane fluidity. In addition to stress responses, CFA biosynthesis also increases as cells enter stationary phase and begin to form sessile biofilms (10, 11, 12, 13), suggesting that CFAs play additional roles in maintaining normal metabolic homeostasis. The cyclopropanation of mycolic acids is required for *Mycobacterium tuberculosis* cell wall structure and impermeability to antibiotics and has long been associated with the virulence of this pathogen and its ability to control host innate inflammation (14, 15, 16). Interestingly, several recent reports have indicated that loss of CFA-containing membrane phospholipids also sensitizes *Helicobacter pylori* to antibiotics (17) and reduces the virulence of *Salmonella enterica* (18), hinting at putative roles for CFAs in bacterial virulence, host interactions, and/or antimicrobial resistance.

Cyclopropane fatty acid synthase (CFAS) is the enzyme responsible for the biosynthesis of CFAs. CFAS is a cytosolic, peripheral membrane protein that transfers a methylene group from SAM across the *cis*-olefin moiety of unsaturated fatty acyl chains within phospholipid bilayers (Fig. 1) (19, 20, 21). The chemical mechanism is believed to proceed through a protonated cyclopropane transition state generated by nucleophilic addition of the olefin moiety of the unsaturated fatty acyl chain onto the electrophilic methyl carbon of SAM, which is rapidly deprotonated by a tightly-bound bicarbonate ion to give the CFA (22, 23, 24, 25). Support for a rate-limiting methyl transfer was provided by kinetic studies of the *Escherichia coli* enzyme using selenium- and tellurium-containing chalcogen analogs of SAM (22). In that study, the elemental effects on the rate of the CFAS-catalyzed reaction paralleled the electrophilicity of the onium congener of the chalcogen, suggesting cleavage of the chalcogen-methyl bond in the transition state. The methyl transfer step was originally thought to be fully rate-limiting (22); however, the deprotonation step was subsequently shown to be at least partially rate-limiting through the measurement of a primary tritium kinetic isotope effect on the cyclopropanation reaction (26). Removal of the active site bicarbonate ion or mutagenesis of the bicarbonate ligands in the CFAS active site also reduced CFA synthase activity, suggesting a direct role for the bicarbonate ion in catalysis (23, 25).

**Figure 1:**
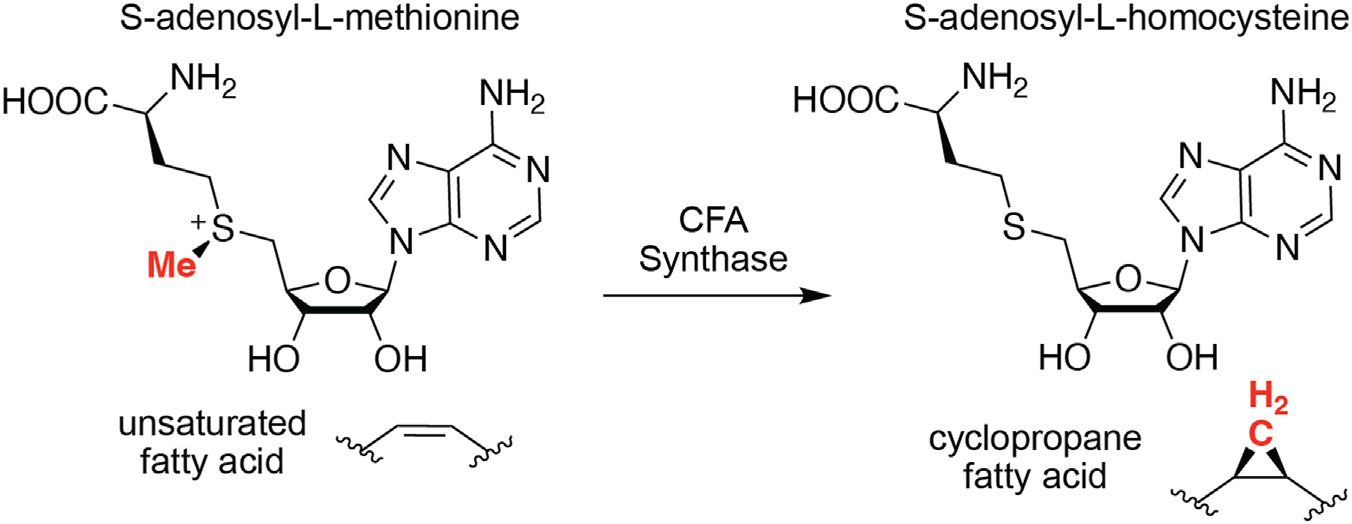
**Reaction catalyzed by cyclopropane fatty acid synthase.** The enzyme transfers an SAM-derived methylene group onto the unsaturated acyl chains of inner membrane phospholipids.

The first crystal structures of enzymes in the CFA synthase family were of the mycolic acid cyclopropane synthases (CmaAs) from *M. tuberculosis* (27). These studies revealed a monomeric enzyme consisting of the Rossmann fold found in many other SAM-dependent methyltransferases (28). In 2018, a crystal structure of the dimeric *E. coli* CFA synthase (EC-CFAS, PDB: 6BQC) was solved by Sauer et al. to a resolution of 2.07 Å. The dimer was subsequently confirmed by sedimentation velocity centrifugation and gel filtration chromatography (29). Distinct from the monomeric *M. tuberculosis* CmaA enzymes, each monomer of EC-CFAS possesses a bi-domain structure, comprised of a small α-helical *N*-terminal domain (NTD) that is absent in the CmaAs and which is attached *via* a flexible linker to the SAM-binding C-terminal catalytic domain. The catalytic domain of the *E. coli* enzyme is highly structurally homologous to the *M. tuberculosis* CmaA enzymes and, similarly, a tightly-bound bicarbonate ion is observed in the EC-CFAS active site, where it interacts with the side chains of Glu239, His266, and Tyr317. Subsequent to this study, high resolution structures were also solved for the *Lactobacillus acidophilus* (PDB 5Z9O) and *Aquifex aeolicus* (7QOS) CFAS enzymes, revealing dimeric structures that strongly resemble the EC enzyme (30). Electron density consistent with a glycerophospholipid was also observed in the EC-CFAS structure. Mass spectrometric analysis of lipids extracted from the EC-CFAS protein crystals suggested that a mixture of several different lipids were present. The acyl chain attached to the *sn*-1 position of the lipid glycerol backbone binds along a hydrophobic pocket of the NTD, whereas the acyl group attached to the *sn*-2 glycerol carbon extends across the domain interface and into the active site of the catalytic domain. Mutagenesis of a small positively charged Arg18/Lys48 patch on the surface of the NTD reduced the apparent vesicle binding affinity in kinetic studies. Collectively, these observations suggest that the NTD plays some role in membrane binding and/or phospholipid extraction from the bilayer and that CFAS may preferentially modify the acyl chain linked to the *sn*-2 position of the glycerol backbone. Mutations to the dimerization interface revealed that monomeric EC-CFAS exhibits a 150-fold lower catalytic efficiency than the WT enzyme. In addition, enzymatic activity significantly dropped when the NTD and catalytic domain were split into separate proteins or when the flexible linker between the two domains was modified in length. These data suggest that protein–protein interactions (both intramolecular and intermolecular) are likely important for proper function of the EC-CFAS dimer on lipid bilayers.

Despite these recent advances, very few CFAS enzymes have been characterized in detail and much remains to be understood regarding the role of CFAs in bacterial adaptation to external stress. In this work, we report the first detailed characterization of the CFAS enzyme from *Pseudomonas aeruginosa* (PA), a ubiquitous Gram-negative bacteria and opportunistic human pathogen responsible for many severe nosocomial infections (31, 32), community-acquired infections among individuals with defects in host defenses (33, 34, 35), and chronic infections in the lungs of cystic fibrosis patients (36). We establish that the PA genome encodes for a single CFAS enzyme and that expression of the *cfas* gene increases during the transition to stationary phase and upon exposure of PA cells to oxidative stress in a manner that depends on the OxyR transcription factor. We next develop a semiquantitative liquid chromatography mass spectrometry (LC-MS) workflow to examine the time-dependent changes in the phospholipid profile of PA strain PAO1 planktonic cultures, where we observe an increase in the abundance of CFAs as the bacteria enter stationary phase. The recombinant PA-CFAS enzyme was then cloned into *E. coli*, expressed, purified, and characterized *in vitro*. Consistent with structural predictions, native ion mobility mass spectrometry reveals a stable PA-CFAS homodimer with each monomer further subdivided into two structurally distinct domains. Using the results from the lipidomic studies as a guide, we conduct a range of *in vitro* kinetic studies of PA-CFAS to study the effects of buffers, ionic strength, and membrane bilayer characteristics on enzyme activity. Overall, our data show that PA-CFAS exhibits preferences for phosphatidylglycerol lipids and membranes with higher levels of fatty acid unsaturation that mimic the late exponential phase membrane composition. Electrostatic interactions between the enzyme and the membrane surface play a modest role in regulating PA-CFAS activity under our conditions. Finally, a phylogenetic analysis of bacterial CFAS enzymes shows that most of the amino acid sequence variation among these enzymes is restricted to the *N*-terminal lipid-binding domain, suggesting that CFAS orthologs may exhibit distinct membrane binding or lipid substrate preferences within their native cellular contexts. The bioinformatic analysis also reveals the presence of a subset of small CFAS enzymes that are truncated at their *C*-termini and lack the structural motifs needed for homodimerization. Cumulatively, this work sets the stage for more detailed mechanistic and biophysical characterization of CFAS enzymes from *Pseudomonads* and other bacterial pathogens, which will help to assess the functional roles of CFAs and whether these important membrane-modifying enzymes are viable targets for antimicrobial development.

## Results

### Identification of PA-CFAS

**Figure 2:**
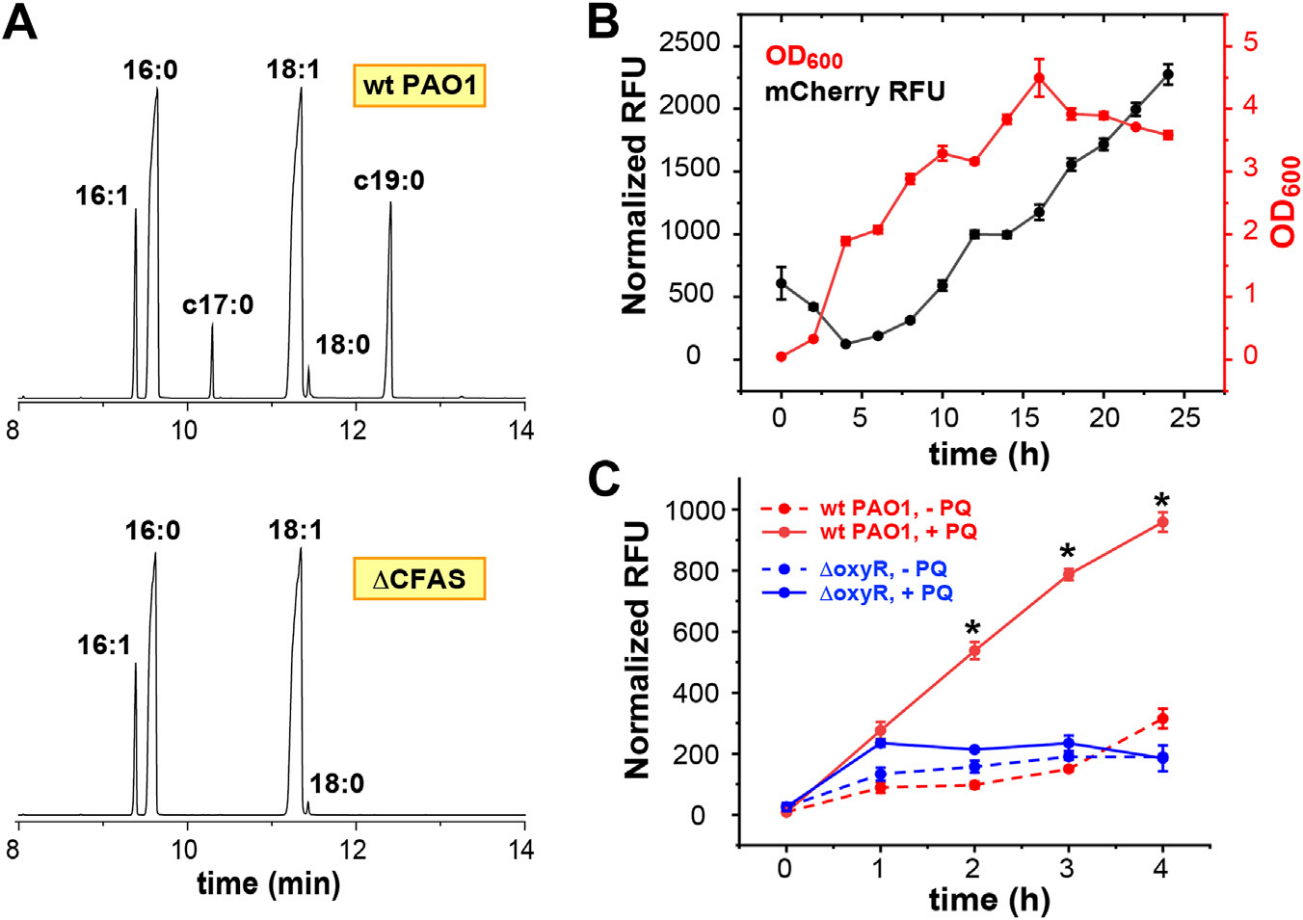
**Validating the function of the *cfa* gene in PAO1.***A*, gas chromatography mass spectrometry (GC-MS) analysis of fatty acids derived from stationary phase cultures of wt PA01 (*top* panel) and the Δ*cfas* KO mutant (*bottom* panel) showing the lack of cyclopropyl fatty acid methyl esters (c17:0 and c19:0) in the Δ*cfas* cells. A quantitative summary of this data is provided in Table S1. *B*, *cfas* gene expression in WT PAO1 was measured using a p_*cfas*_-mCherry reporter activity during growth in LB medium (data shown as mean ± SD, n = 3 biological replicates). *C*, *cfas*-mCherry reporter activity in stationary phase WT and *oxyR* mutant strains challenged with 1.25 mM paraquat (PQ) (added at T = 0 h) or water as control (data shown as mean ± SEM, n = 9 biological replicates pooled from three independent experiments). ∗*p*-value <0.0001 using the Mann-Whitney test compared to the *oxyR* mutant in the presence of 1.25 mM paraquat. In panels B and C, the relative fluorescence units (RFU) were calculated from the emission at 620 nm (with 587 nm excitation) normalized by the bacterial biomass measured by the absorption at 600 nm (A_600_).

Although *P. aeruginosa* is known to produce CFAs, no CFAS enzyme has been genetically identified and biochemically characterized to date. Thus, we scanned the PAO1 genome, performed a Conserved Domain Search for a class I SAM-dependent methyl transferase domain and identified a single gene (PA5546) encoding a conserved hypothetical protein from the PF02353 family (InterPro ID = IPR003333) that shared 42.8% amino acid sequence identity with EC-CFAS. Among 7982 PA genomes available (www.pseudomonas.com), the PAO1 PA5546 coding sequence had 100% nucleotide identity to 321 genomes and ≥ 99.4% nucleotide identity to 4000 genomes, suggesting a highly conserved gene in this species. To confirm that *cfas* was the CFA synthase in PA, we generated an isogenic unmarked in-frame deletion mutant of PA5546 in the PAO1 strain by allelic exchange. Fatty acid methyl ester analysis of lipids extracted from stationary phase cultures of these strains by gas chromatography mass spectrometry (GC-MS) confirmed that the Δ*cfas* mutant produced no detectable CFAs as compared to the WT isogenic parental strain (Fig. 2*A* and Table S1).

Since CFAs alter the biophysical properties of lipid bilayers, including an increase in the bacterial inner membrane fluidity (7), we used 6-lauroyl-2-(dimethylamino)-naphtalene (Laurdan), a well-established membrane fluorescent probe sensitive to membrane lipid packing (37, 38), to compare the WT and isogenic Δ*cfas* mutant. The Laurdan generalized polarization (${GP}_{ex}$) of WT stationary phase cells (${GP}_{ex}=0.43\pm 0.03$) decreased to a value of 0.37±0.02 (P=0.01) in the Δ*cfas* mutant, indicating increased membrane fluidity upon deletion of *cfas*. Both unsaturated fatty acids (the substrates of CFAS) and CFAs disrupt membrane packing and increase fluidity relative to the saturated fatty acids found in bacterial membranes (7). However, our GC-MS analysis of fatty acid content (Fig. 2*A* and Table S1) suggests a similar fractional abundance of saturated fatty acids (16:0 and 18:0) in both the wt PAO1 (39.3%) and in the Δ*cfas* KO strain (42.1%). Thus, the increased fluidity measured by the Laurdan assay for the wt cells is likely attributable to the CFA content of these cells. Nevertheless, the precise role of CFAs and unsaturated fatty acids on bacterial membrane properties remain equivocal and are likely complex and condition-dependent (7).

### *Cfas* gene expression profile and regulation

Previous studies in *E.coli* and *Pseudomonas putida* have shown that *cfas* gene expression is induced during stationary phase (10, 39). In order to profile *cfas* gene expression in PA over time, we constructed a *cfas* promoter—mCherry fusion (p_*cfas*_-mCherry)—to serve as a transcriptional reporter. Using this construct, we confirmed that *cfas* gene expression is also strongly induced in WT PAO1 starting in mid-exponential phase and upon transition to stationary phase during planktonic growth in LB medium (Fig. 2*B*). Interestingly, the sequence upstream of the *cfas* coding region also contained an OxyR-binding site, suggesting that *cfas* may be regulated by the oxidative stress regulator OxyR and, as such, may be induced under oxidative stress conditions. To test this, we measured p_*cfas*_-mCherry reporter activity in PAO1 cultures challenged with paraquat, a redox-cycling superoxide-generating compound known to strongly activate OxyR-dependent responses (40). We observed that p_*cfas*_-mCherry activity was induced 5-fold (4 h post-challenge) in WT PAO1 upon exposure to sublethal concentrations of paraquat. In contrast, this paraquat-dependent induction of the *cfas* gene is abrogated in the Δ*oxyR* mutant (Fig. 2*C*), suggesting that *cfas* expression is indeed OxyR-dependent under these conditions.

### Characterizing the phospholipid composition of *P. aeruginosa* membranes and CFAs at different growth phases during planktonic growth

**Figure 3:**
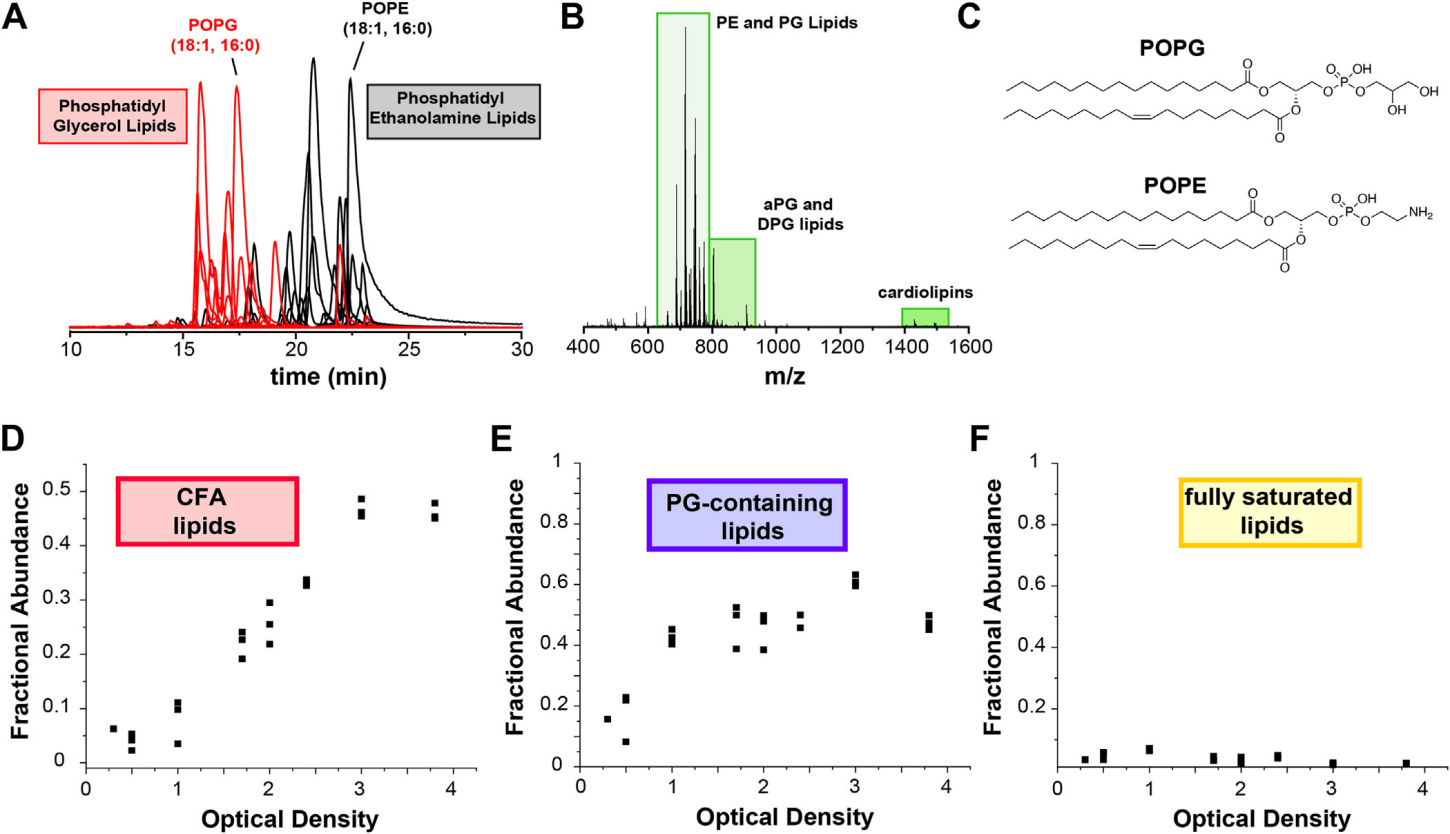
**Characterization of PAO1 total phospholipid extract by liquid chromatography mass spectrometry.***A*, extracted ion chromatograms for the phosphatidylethanolamine (PE, *black*) and phosphatidylglycerol (PG, *red*) lipids analyzed in this study. The data shown are derived from a wt PAO1 extract harvested at an optical density (600 nm) of 1.7. Additional analysis and validation of these lipids are provided in Figs. S1–S4 and Table S2 of the Supporting Information. *B*, mass spectrum of compounds eluting in the 15 to 25 min time window. In addition to the abundant PE and PG lipids, alanyl-phosphatidylglycerol (aPG) lipids, diphosphatidylglycerol (DPG) lipids, and cardiolipins were detected (Table S2). *C*, the chemical structures of the commercially available POPG and POPE lipids used in this study. Also shown are the time-dependent changes in the fractional abundances of (*D*) lipids containing cyclopropanated fatty acids, (*E*) lipids containing a phosphatidylglycerol (PG) head group, and (*F*) lipids containing two fully saturated acyl chains.

Since *cfas* gene expression is induced upon entry into stationary phase, we employed an LC-MS–based workflow to profile the time-dependent changes in the membrane phospholipid composition of PAO1 cells as the growing cultures progress from exponential to stationary phase. Accordingly, planktonic cultures of WT PAO1 were grown in LB medium and were harvested at optical densities ranging from early exponential phase (A_600_ = 0.3) to late stationary phase (A_600_ = 3.8). Total lipids were then extracted from the cell pellets using a Bligh-Dyer protocol (41) and were analyzed by C18 reverse phase LC-MS. We analyzed a total of 29 different phosphatidylethanolamine (PE) and phosphatidylglycerol (PG) lipids (Fig. 3), which represent the two most abundant phospholipid classes in PA and most other bacteria, and are known to comprise the majority of PAO1 inner membranes (42, 43, 44, 45, 46). The identities of the PE and PG lipids were validated by collision-induced dissociation fragmentation and tandem mass spectrometry analysis (Figs. S1-S4, Table S2). Consistent with previous studies on *P. aeruginosa* (46), we also detected a number of cardiolipins, diphosphatidylglycerol lipids, and alanyl-phosphatidylglycero006C lipids (Table S2), but these lipids were present at lower abundance (Fig. 3*B*) and were not analyzed further in this study. Although phospholipids and lipopolysaccharides are the major lipid components of bacterial membranes, diacylglycerols are produced in low quantities in bacterial membranes as by-products of several enzymatic reactions (46, 47) and certain bacteria accumulate triacylglycerols as energy storage molecules under specific growth conditions (48). Moreover, under phosphate-limiting conditions, some bacteria can convert membrane phospholipids into a variety of phosphorous-free lipids (such as ornithine lipids and various glycolipids) (46). However, to our knowledge, direct cyclopropanation of unsaturated acyl chains found within diacylglycerols, triacylglycerols, and other phosphate-free bacterial lipids by a CFAS enzyme has not yet been demonstrated, and none of these lipid classes were detected in our analysis.

Control experiments indicated that PE and PG lipids have similar ionization efficiencies under our LC-MS conditions (Fig. S5). Thus, we performed a semiquantitative analysis to estimate the fractional abundances of lipids detected in the total lipid extract of WT PAO1 (Fig. 3, *D*–*F*). We then profiled the time-dependent changes in lipid fractional abundance to track the formation of CFAs as the PA cells entered stationary phase and to approximate the membrane composition that is most suitable for assessing *in vitro* PA-CFAS activity. Consistent with the time scale of *cfas* gene expression (Fig. 2*B*), the major cyclopropane containing lipids (PE 33:1, PE 33:2, PE 35:1, PE 35:2, PG 33:1, PG 35:1, PG 35:2) began to accumulate in early stationary phase (around A_600_ = 1.0) and continued to increase to a combined fractional abundance of approximately 45% of the total extract (Fig. 3*D*). This value is similar to total CFA quantities measured previously in WT PAO1 lipid extracts (42). Also consistent with previous studies, the ratio of PE:PG lipids under our growth conditions was maintained at approximately 1:1 beginning in late exponential phase (Fig. 3*E*), and the majority of lipids detected possessed at least one unsaturated acyl chain (Fig. 3*F*). These data are in excellent agreement with the high fractional abundance of unsaturated fatty acids detected in our GC-MS analysis (Fig. 2*A* and Table S1) and suggest that an optimal membrane composition for PA-CFAS activity would have roughly equivalent amounts of each PE and PG head group as well as a high abundance of unsaturated substrate acyl chains.

### Cloning, expression, and purification of PA-CFAS

**Figure 4:**
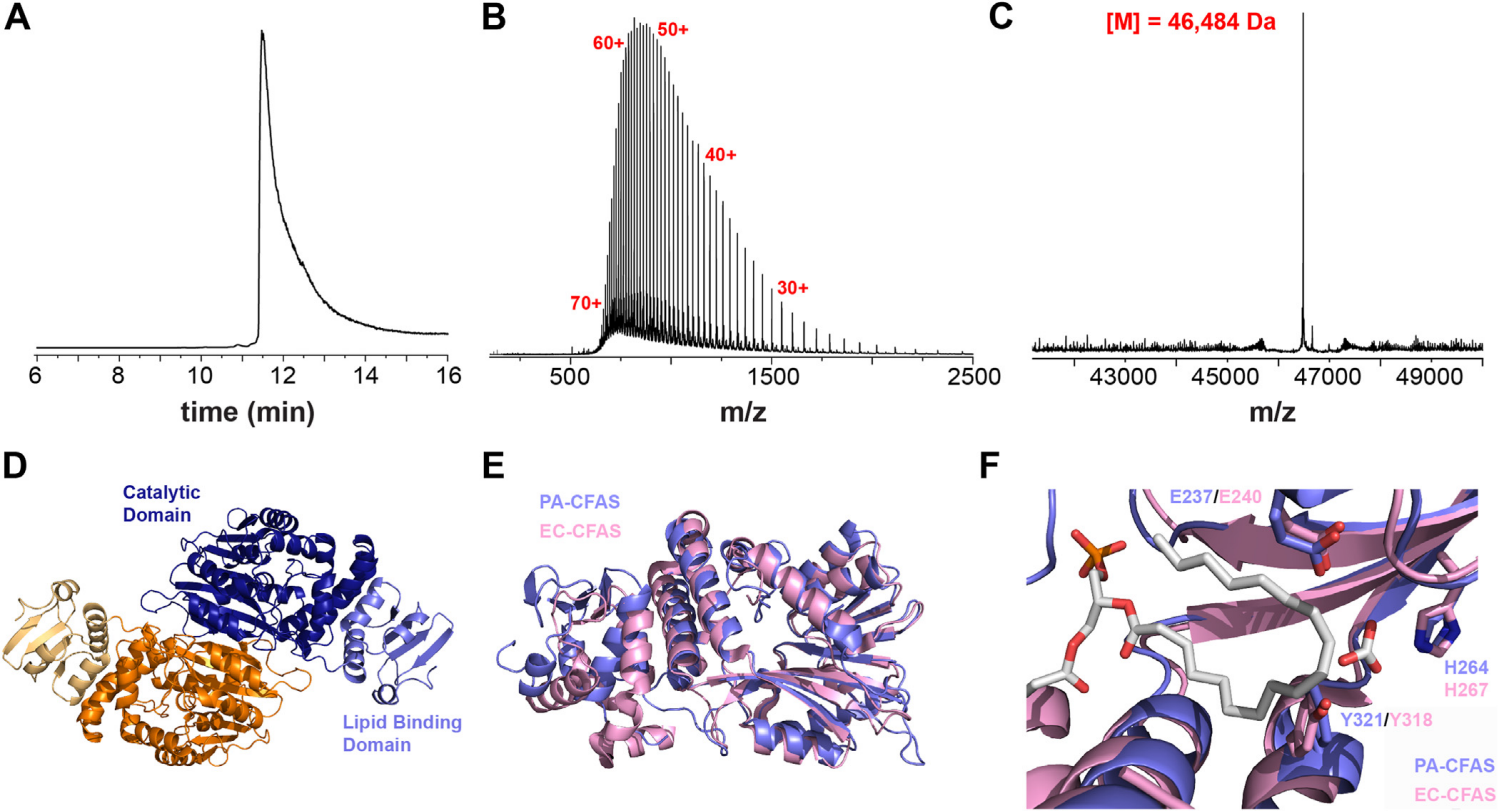
**Heterologous expression, purification, and structural prediction of PA-CFAS.***A*, total ion chromatogram of PA-CFAS as analyzed by C4-reverse phase LC-MS following purification of the protein by Ni-NTA affinity and size-exclusion chromatography. *B*, electrospray ionization (ESI) mass spectrum of the chromatographic peak from (*A*), where PA-CFAS was observed in charge states ranging from approximately 20 to 75^+^. *C*, the deconvoluted mass spectrum from (*B*) indicates the presence of a molecule with a molecular weight that exactly matches the expected molecular weight of His_6_-tagged PA-CFAS. A similar analysis was performed on the other enzymes employed in this study (Fig. S13). *D*, AlphaFold multimer model of the PA-CFAS dimer. The individual monomers are colored in *blue* and *orange*. The catalytic domain and lipid-binding domains are shaded in dark and light tones, respectively. *E*, structural alignment of the PA-CFAS AlphaFold model (*blue*) with the experimentally determined EC-CFAS X-ray crystal structure (*pink*). The RMSD for this alignment was 1.239 Å over 1551 atoms. The majority of the deviation occurs in the *N*-terminal lipid-binding domain. A small segment of the PA-CFAS (A91-L113) located between the lipid-binding and catalytic domains has been removed for clarity. The analogous region was not observed in the EC-CFAS crystal structure and is likely disordered. *F*, close view of the PA-CFAS active site, showing structural conservation of the ligands to the catalytic bicarbonate ion and the phospholipid substrate observed in the EC-CFAS crystal structure.

To examine the biophysical properties and biochemical function of PA-CFAS, the recombinant enzyme was cloned as a His_6_-fusion protein, heterologously expressed in *E. coli*, and purified to near homogeneity using Ni-affinity and gel filtration chromatography. An analysis of the purified protein using LC-MS shows a highly pure preparation at the exact molecular weight expected for the PA-CFAS monomer (${MW}_{calc}=$ 46,486 Da, ${MW}_{obs}=$ 46,486 Da, Figure 4, *A*–*C*). The purification procedure yielded approximately 5 ml of 70 μM soluble protein per 3 L of LB culture. The *Pseudomonas* CFAS enzyme (PA-CFAS) contains all of the conserved residues necessary for SAM binding and cyclopropanation catalysis, as well as the three conserved bicarbonate ligands (Glu237, His264, and Tyr321). AlphaFold multimer predicted a dimeric structure for PA-CFAS that is nearly superimposable with the X-ray crystallographic model of the EC-CFAS enzyme (Fig. 4, *A*–*C*). Overall, these data suggested that PA-CFAS is likely a dimer that possesses a topological structure very similar to its *E. coli* ortholog.

### Native ion mobility mass spectrometry characterization of PA-CFAS

**Figure 5:**
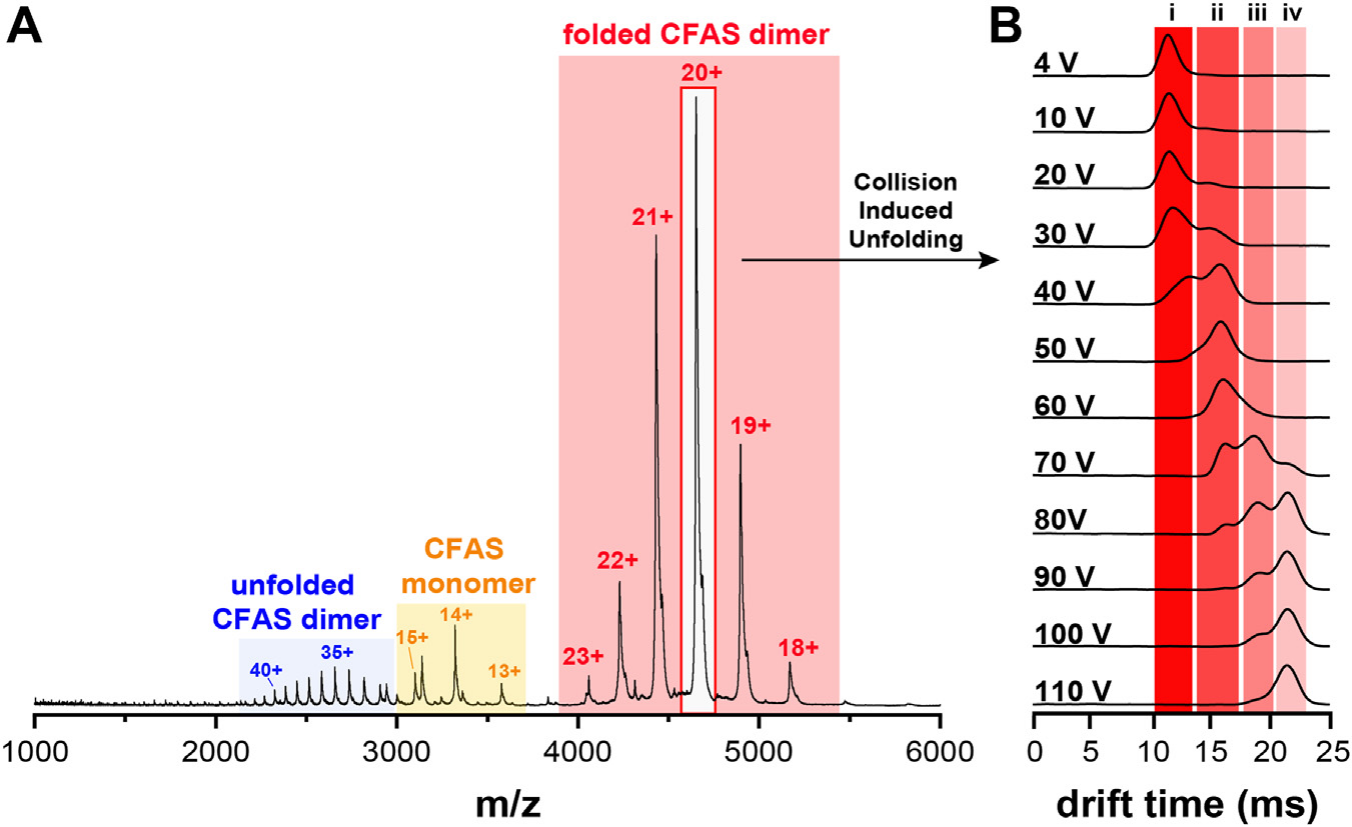
**Native mass spectrometry characterization of PA-CFAS.***A*, the native mass spectrum of the PA-CFAS dimer reveals a bimodal charge state distribution that indicates the presence of two major gas phase conformations. The well-folded population at higher m/z (charge states 18–23^+^) is more abundant and carries fewer charges because the folded enzyme exposes fewer sites for charge transfer during the electrospray process. *B*, collision-induced unfolding analysis of the 20^+^ ion of the PA-CFAS dimer. As the trap collision energy is increased from 4 to 110 V, the native conformation (*top*, species i) unfolds through two intermediates (ii and iii) into a fully unfolded conformation (species iv). The observation of the three unfolding transitions is consistent with the expected domain architecture of the PA-CFAS dimer (Fig. 4*D*).

To perform a more detailed structural analysis of the PA-CFAS enzyme, we characterized the enzyme using native ion mobility mass spectrometry, a technique that allows the tertiary and quaternary structure of natively folded protein complexes to be examined in the gas phase environment of the mass spectrometer (Fig. 5). The native mass spectrum revealed a bimodal PA-CFAS dimer population, characterized by a folded population (the 18–23^+^ charge states) as well as a partially unfolded, more highly charged conformation (the 31–41^+^ charge states). The molecular weights measured from the 18 to 23^+^ charge states (93,030 Da) and the 31 to 41^+^ charge states (92,972 Da) are in close agreement with the theoretical mass of the dimer (92,972 Da). The slightly increased mass determined from the 18 to 23^+^ charge states is likely due to incomplete desolvation of the folded CFAS dimer under the gentle nanoESI conditions employed. We next calculated the solvent accessible surface area of the PA-CFAS dimer from the bimodal charge state distribution using a well-established empirical relationship (49, 50, 51). Using the 18 to 23^+^ and 31 to 41^+^ charge states, we calculated surface areas for the two CFAS dimer conformations of 31,700 ± 200 Å^2^ and 81,900 ± 400 Å^2^, respectively. The solvent accessible surface area calculated for the AlphaFold model of the PA-CFAS dimer (32,823 Å^2^) is in excellent agreement with the surface area calculated from the 18 to 23^+^ ions. This data strongly suggests that the PA-CFAS dimer exists in a near native conformation under our native MS conditions. Interestingly, despite the apparent partial unfolding of the CFAS dimer into a more open and highly charged conformation (*i.e*. the 31–41^+^ ions observed in the spectrum), the dimer remains intact. This suggests that the intermolecular protein–protein interactions at the dimer interface are relatively strong and survive both the ionization conditions in the nano-electrospray ionization (ESI) source as well as the gas phase transit from the source to the detector. Homodimerization has been shown to strongly stimulate the activity of the *E. coli* CFAS enzyme (29).

Finally, we characterized the conformational landscape of the natively folded PA-CFAS dimer using ion mobility mass spectrometry, a method that separates gas phase ions on the basis of their shapes (Fig. 5*B*). Analysis of the near natively folded 20^+^ ion of the PA-CFAS dimer at low collision energy (4 V) revealed a homogeneous population of ions with a narrow shape distribution that elutes from the ion mobility cell at early drift times. This behavior is consistent with a well-folded and conformationally compact PA-CFAS dimer that exists in a single major conformational state. As the collision energy (applied in the ion path prior to ion mobility separation) was gradually increased, the PA-CFAS dimer began to unfold through a series of three, well-defined conformational populations that elute at subsequently later drift times, consistent with the stepwise unfolding of the dimer. Similar gas phase collision–induced unfolding experiments have shown that multidomain-containing proteins typically unfold in a stepwise process, undergoing one unfolding event for each domain of the protein (52). Thus, the three-step collision-induced unfolding transition for PA-CFAS is consistent with the predicted structural model and domain topology of the PA-CFAS dimer, consisting of two *N*-terminal lipid-binding domains and two C-terminal catalytic domains. The amenability of PA-CFAS toward native ion mobility MS characterization will enable future studies to investigate the effects mutagenesis and of lipid and ligand binding on the conformational landscape and dynamics of the enzyme, which are likely important for biochemical function (29).

### Biochemical characterization of PA-CFAS

To study the kinetic properties of PA-CFAS, we adapted a colorimetric assay developed previously for EC-CFAS by Guianv’rc'h, where the SAH produced by CFAS catalysis is enzymatically converted into homocysteine by the combined action of SAH nucleosidase and LuxS (Fig. S6) (53). The homocysteine can then be quantified spectrophotometrically by reaction with Ellman’s reagent (5,5′-dithio-bis[2-nitrobenzoic acid], DTNB). To ensure that the coupled assay was working properly in our hands, we first determined the steady state kinetic parameters for the *E. coli* CFAS with SAM as the variable substrate in HEPES buffer at pH 7.5 using 100 nm unilamellar liposomes composed of 1,2-dioleoyl-phosphatidylethanolamine (PE 18:1/18:1) and 1,2-dipalmitoyl-phosphatidylglycerol (DPPG, PG 16:0/16:0) in a molar ratio of 60:40. Under these conditions, we obtained similar steady state kinetic parameters to values reported previously for the EC-CFAS enzyme ($k_{cat}=13\;\min^{-1},K_{m,SAM}=68\;\mu M$, Fig. S7) (22, 25).

**Figure 6:**
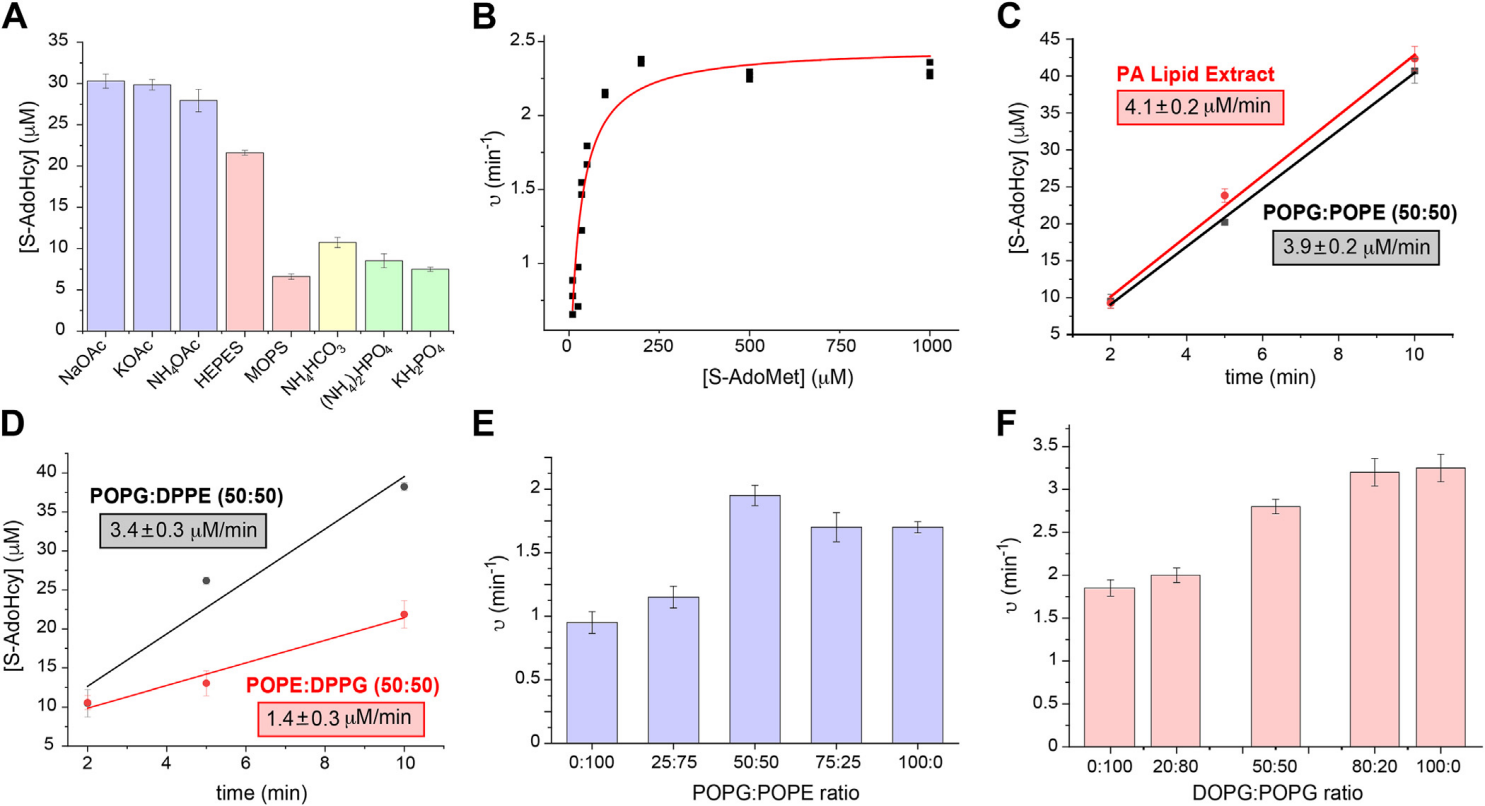
**Biochemical and kinetic analysis of PA-CFAS.** All assays contained 2 μM PA-CFAS, 1 mg/ml small unilamellar vesicles, 2 μM SAHNase, and 10 μM LuxS at pH 7.5 and were conducted at 37 °C. The reactions in panels B-F were conducted in 200 mM KOAc. The reactions in panels A and C-F were conducted with 1 mM SAM. *A*, dependence of PA-CFAS activity on various buffers and ionic salts. End point assays were conducted for 20 min in 200 mM of the indicated buffer/salt with 50:50 POPE:POPG vesicles. *B*, a steady state kinetic analysis of PA-CFAS in 200 mM potassium acetate using 50:50 POPE:POPG vesicles and SAM as the variable substrate yielded $k_{cat}=2.5\pm 0.08\;\min^{-1}$ and $K_{m,SAM}=26\pm 3\;\mu M$. *C*, comparison of PA-CFAS activity in 200 mM potassium acetate using vesicles prepared either from a 50:50 mixture of POPE:POPG or from a PA phospholipid extract harvested in exponential phase (A_600_ = 0.7). *D*, PA-CFAS exhibits higher activity with PG lipid substrates (as opposed to PE substrates) in membranes with identical head group and acyl chain compositions. *E*, dependence of PA-CFAS activity on membrane head group composition. The trend mimics the observed preference for PG substrates observed in panel D. *F*, dependence of PA-CFAS activity on the extent of acyl chain unsaturation in PG membranes. PA-CFAS activity increases with increasing DOPG (dioleoyl-phosphatidyl glycerol) as the extent of acyl chain unsaturation increases.

For profiling PA-CFAS activity, we chose to employ mixtures of 1-palmitoyl-2-oleoyl-phosphatidyl ethanolamine (POPE, PE 16:0/18:1, a zwitterionic lipid) and 1-palmitoyl-2-oleoyl-phosphatidyl glycerol (POPG, PG 16:0/18:1, an anionic lipid), each of which contains one fully saturated C16-palmitoyl chain in the *sn*-1 position of the glycerol backbone and a singly-unsaturated C18-oleoyl chain in the *sn*-2 position (Fig. 3*C*). POPE and POPG are also two of the most abundant PAO1 membrane phospholipids detected in our lipidomic studies (Fig. 3*A*). Using anionic vesicles (50%:50% POPE:POPG) that mimic the head group composition of late exponential phase PAO1 membranes, we screened for activity at pH 7.5 in a variety of different buffers and ionic salts using a 20 min end point assay (Figs. 6*A* and S8). CFAS activity was detected in each case, but the highest activities were obtained in the reactions containing 200 mM acetate salts. In general, the CFAS activity decreased at both higher and lower ionic strengths (Fig. S8). The steady state kinetic parameters determined for the PA-CFAS enzyme in 200 mM potassium acetate provided slightly lower values for $k_{cat}\;\left(2.5\pm 0.08\;\min^{-1}\right)$ and $K_{m,SAM}$ (26±3μM) than those measured for the EC-CFAS enzyme, but the overall catalytic efficiencies ($k_{cat}/K_{m,SAM}$) of the two enzymes were similar (Figs. 6*B* and S7). To test whether our synthetic POPE:POPG vesicles supported similar levels of PA-CFAS activity as native PA membranes, we also prepared vesicles from a phospholipid extract derived from exponentially growing PAO1 cells. This extract yielded a nearly identical initial rate as the 50:50 POPE:POPG vesicles (Fig. 6*C*), suggesting that our synthetic membranes are likely a suitable mimic for the native PA-CFAS substrate under these *in vitro* conditions.

To determine whether the PA-CFAS active site exhibits any preference for the identity of the lipid head group, we conducted a comparative initial rate assay with vesicles containing identical head group compositions (50:50 PG:PE) and identical fractional abundances of unsaturated fatty acyl chains (25%), but with the unsaturated acyl chain substrate linked to different backbones (either PG or PE). Namely, we compared the initial rate over a 10 min time interval with vesicles composed of either 50:50 POPG:DPPE (where DPPE = dipalmitoyl phosphatidylethanolamine, PE 16:0/16:0) or 50:50 POPE:DPPG (where DPPG = dipalmitoyl phosphatidylglycerol, PG 16:0/16:0). The data indicate a clear preference for the anionic POPG lipid over the zwitterionic POPE as a substrate for PA-CFAS (Fig. 6*D*). Given that the membrane surface charge, acyl chain lengths, and unsaturated acyl chain abundances are identical in these two vesicle preparations, the data suggest that PA-CFAS likely makes specific intermolecular binding interactions with the PG head group that favor preferential modification of PG-linked unsaturated acyl chains. The structural basis for this specificity is currently unknown and will require further investigation.

To test whether membrane surface charge plays any role in regulating PA-CFAS activity, we also varied the relative concentrations of POPE (zwitterionic) and POPG (anionic) lipids, which allowed the net anionic character of the model membranes to be modulated without altering the fractional abundance of unsaturated fatty acids or acyl chain lengths within the bilayer. Accordingly, the initial rate of PA-CFAS activity was measured over a 10 min interval with 1 mM SAM at pH 7.5 in 200 mM potassium acetate (Fig. 6*E*). The data show a preference for vesicles containing higher quantities of PG, but the effect was modest, and may simply reflect the preference for PG lipids illustrated in Figure 6*D*. These data suggest that electrostatic interactions do not strongly govern CFAS activity under these *in vitro* conditions, a finding that is consistent with the modest effects of ionic strength on PA-CFAS activity (Fig. S8).

Finally, our semiquantitative lipidomic studies suggested that PA membranes are highly enriched in unsaturated fatty acids (Fig. 3*F*). To determine to what extent acyl chain packing in the bilayer and membrane fluidity affect PA-CFAS activity, we conducted experiments within 100% PG membranes and varied the fractional abundance of unsaturated acyl chains by altering the ratio of POPG (which contains a single unsaturated oleoyl acyl chain) and DOPG (dioleoylphosphatidyl glycerol, PG 18:1/18:1), which contains two oleoyl acyl chains. This experiment revealed a systematic increase in the initial velocity of the CFAS-catalyzed reaction as the proportion of DOPG increased. The activity on 100% DOPG membranes is approximately twice the activity observed on 100% POPG membranes. Thus, it appears that PA-CFAS activity is influenced by the extent of acyl chain packing and fluidity within the lipid bilayer. The enhanced activity of PA-CFAS on more highly unsaturated membranes would be consistent with a role for this enzyme in reducing the permeability of membranes in response to acid shock or chemical perturbations.

### Bioinformatic analysis of CFAS

**Figure 7:**
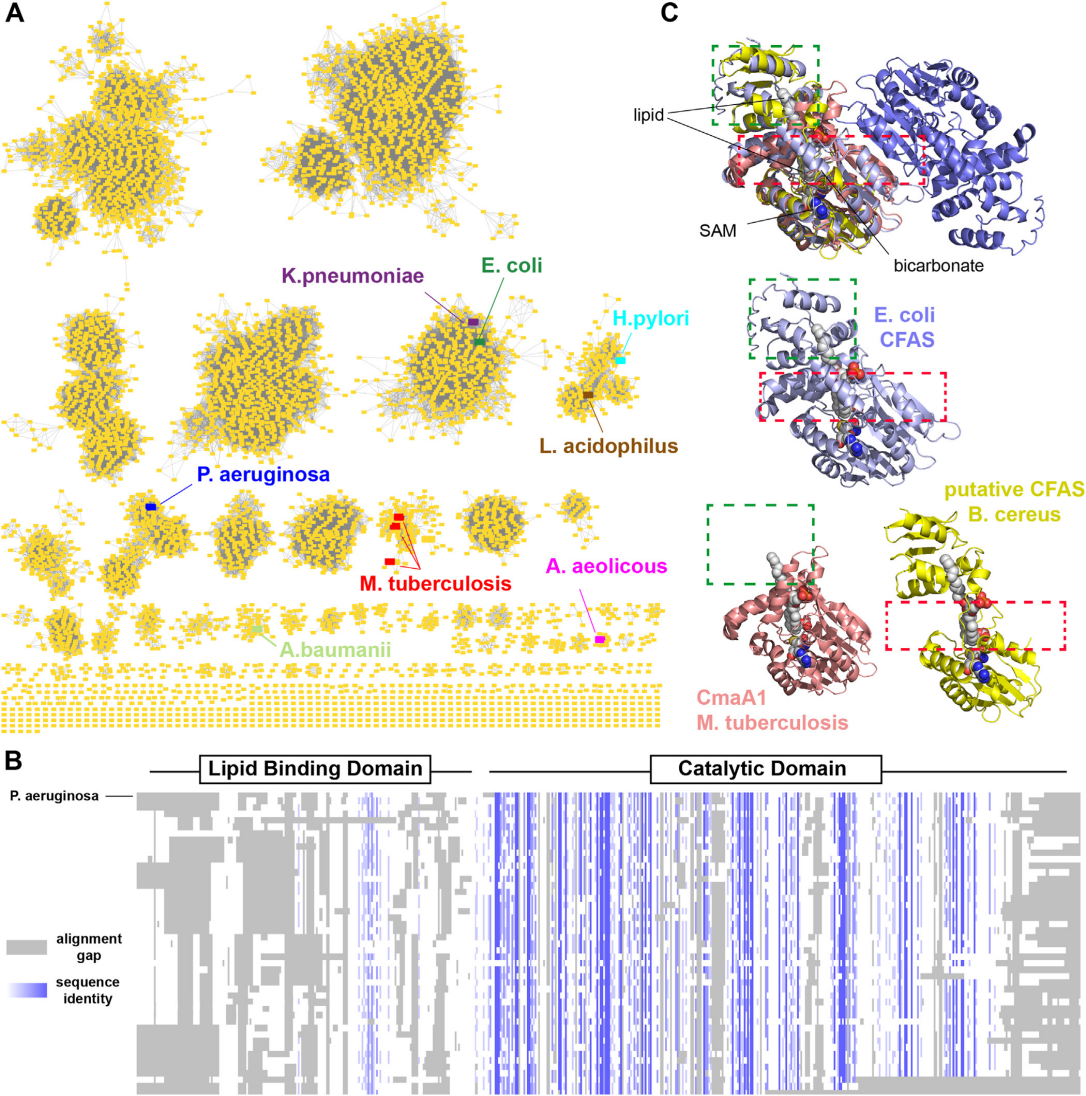
**Bioinformatic analysis of the CFAS enzyme family.***A*, sequence similarity network for bacterial cyclopropane fatty acid synthases. The network was generated by the Enzyme Function Initiative server using the Interpro identifier for the CFAS family (IPR003333) as the query and a bacterial taxonomic filter, resulting in a total of 31,857 unique sequences. The final network was generated with an alignment score of 110 and enzymes sharing greater than 50% sequence identity were grouped into single nodes. CFAS enzymes that have been biochemically or structurally characterized are highlighted in the network, along with enzymes from several human pathogens. *B*, sequence alignment of 50 CFAS enzymes (sequence length 350–500 amino acids) selected randomly from the network are compared to the PA-CFAS enzyme. More highly conserved sequences are colored in darker shades of *blue*. Gaps in the alignment are shaded *gray*. The catalytic domain is more highly conserved whereas the lipid-binding domain is more variable. *C*, shown at the top is a structural alignment of EC-CFAS (pdb 6BQC) (29), the CmaA1 enzyme from *M. tuberculosis* (pdb 1KPG) (27), and the AlphaFold model of a small CFAS form *Bacillus cereus* (Uniprot ID = A0A164GXD1). The individual proteins are shown below in the same orientation. CmaA1 lacks the *N*-terminal lipid-binding domain (*green* box). The *B. cereus* enzyme lacks the dimerization and intramolecular interaction interfaces (*red* box). The putative binding sites for the phospholipid substrate, *S*-adenosyl-l-homocysteine product, and active site bicarbonate ion are shown in space filling orientation. Additional representations of the network and sequence alignments are provided in Figs. S9–S11.

To gain a better understanding of the phylogenetic distribution and structural homology of CFAS enzymes, we constructed a sequence similarity network using the Enzyme Function Initiative – Enzyme Similarity Tool (Fig. 7*A*) (54, 55). Using the conserved mycolic acid cyclopropane synthase domain (PF02353, IPR003333) as the query, we retrieved a total of 33,466 unique sequences. Of these sequences, 4.8% were found in eukaryotes and the remaining 31,857 enzymes (95.2%) were spread across 38 bacterial phyla; however, the *Pseudomonadota* (including *E. coli* and *P. aeruginosa*) and the *Actinomycetoma* (including the mycobacteria) account for the large majority of these enzymes (57.5% and 26.5%, respectively, Fig. S9). In the vast majority of enzymes that contain the PF02353 conserved domain (>97%), the PF02353 domain is not fused to any other protein domains and appears to function as a standalone enzyme. The amino acid sequence length distribution histogram (Fig. S10) shows that CFAS enzymes fall into two main groups. Approximately 86% of the sequences are proteins of length 350 to 500 amino acids. This group includes the *E. coli* and *P. aeruginosa* enzymes, as well as CFAS homologs from other important human pathogens such as *H. pylori*, *Acinetobacter baumannii*, *Klebsiella pneumoniae*, and *Enterococcus faecalis*. Among this group, the *C*-terminal catalytic domain is more highly conserved, whereas the putative *N*-terminal lipid-binding domain is more highly variable (Fig. 7*B*). The weakly conserved portion of the lipid-binding domain (EC-CFAS residues Leu57-Trp68) maps to the interface with the catalytic domain in the EC-CFAS X-ray crystal structure.

A smaller subpopulation (about 10%) of enzymes in the network are proteins of sequence length 280 to 320 amino acids that are distributed throughout the network (Fig. S11). These small CFAS sequences fall into two separate groups. Approximately 80% of the small CFASs lack the putative *N*-terminal lipid-binding domain but possess an intact *C*-terminal catalytic domain (Fig. 7*C*). This group includes the three mycobacterial cyclopropane mycolic acid synthase (CmaA) enzymes whose structures have been solved (27). These enzymes function on the unsaturated precursors of the mycolic acid moieties found in the mycobacterial cell envelope, which are not embedded in the plasma membrane. This suggests that the *N*-terminal lipid-binding domain of PA-CFAS (and its homologs) serves a function that is specific to lipid substrates located in the plasma membrane. Interestingly, the remainder of the small CFAS enzyme appears to possess an intact *N*-terminal lipid-binding domain and a *C*-terminally truncated catalytic domain. These enzymes lack one of the conserved active site bicarbonate ligands (Tyr318 in *E. coli*) as well as the homodimerization interface (*E. coli* Ser294-Phe314) and a large α-helix (*E. coli* residues E341-A361) that forms part of the interface between the *N*- and *C*-terminal domains. The role of these small CFAS enzymes is not known, but the essential SAM-binding residues are retained, suggesting that these enzymes may catalyze methylation or cyclopropanation of substrates located at the bacterial membrane. The known *N*-methyltransferases that convert PE lipids into phosphatidylcholines in bacteria fall into a distinct family of SAM-dependent enzymes (PF13649, IPR041698) (56), suggesting that the small CFASs would likely perform a separate function.

## Discussion

Bacterial membranes serve as the first line of defense against antimicrobials. A better mechanistic understanding of bacterial membrane biology and metabolism is needed to identify new potential targets and pathways for novel antimicrobial development. One such potential target are the bacterial CFAS enzymes, which restructure bacterial membranes during the exponential to stationary phase transition and in response to a variety of external cues (4). The cyclopropane fatty acyl–containing lipids produced by CFAS enable bacteria to modulate the permeability of their plasma membranes in response to changes in extracellular pH, changes in temperature, and/or the presence of chemical perturbations. In *E.coli*, CFAS is regulated at multiple levels, most notably at the transcriptional level *via* RpoS- and ppGpp-dependent mechanisms but also through activating and repressing small RNAs (57) and RpoH-dependent proteolytic degradation (58). CFAs accumulate naturally as bacterial cells undergo the major metabolic transition from rapidly dividing single cells into stationary phase and biofilm formation, and several recent studies suggest that CFA-containing lipids can contribute to infections caused by *H. pylori* and *S. enterica* (17, 18). While widespread across many bacterial phyla, CFAs are more prevalent in the inner membranes of gram-negative bacteria, a class of bacteria with a structurally complex cell envelope for which novel antimicrobials are urgently needed. In this study, we report the first detailed characterization of CFA biosynthesis and of the CFAS enzyme in *P. aeruginosa*, an opportunistic Gram-negative pathogen that causes many acute infections as well as chronic, biofilm-associated infections in humans. Management of PA infections has become increasingly more difficult due to the prevalence multi-drug resistant strains (59).

In this study, we located the *cfas* gene in the PAO1 strain and demonstrated through genetic manipulation of the parental strain that this highly conserved gene encodes the sole enzyme responsible for CFA biosynthesis. As has been observed in both *E. coli* and *P. putida*, transcription of the *cfas* gene in PAO1 increases as planktonic cells transition into stationary phase. Complementary GC-MS and LC-MS analyses show that this growth phase–dependent increase in the CFAS enzyme indeed correlates with increases in both CFAs and CFA-containing membrane phospholipids. Interestingly, we demonstrate that *cfas* transcription in PAO1 is additionally regulated by OxyR in response to oxidative stress, adding to the growing body of evidence suggesting that CFA biosynthesis in bacteria is a general response to many different types of external stress. In light of this finding, a more thorough analysis of the promoter regions of bacterial *cfas* genes could provide additional insights into the factors that regulate CFA biosynthesis. At the cellular level, the effects of CFA accumulation on membrane biophysics, trans-membrane transport, and the functions of integral membrane proteins and protein complexes all remain open questions that will need to be investigated in order to better understand bacterial adaptation to harsh environments, such as those typically encountered by human pathogens.

We also developed a semiquantitative LC-MS–based lipidomic analysis to profile time-dependent changes in PAO1 membranes. This method should prove to be a useful tool for profiling the lipid content of different PA strains, mutants, or clinical isolates, and/or for investigating the effects of growth conditions or chemical perturbations on the membrane composition of PA or other bacterial species of interest. Guided by results obtained from the lipidomic analysis, we designed assays to investigate the *in vitro* biochemical properties of PA-CFAS. Under optimal *in vitro* conditions, the PA-CFAS enzyme exhibited a catalytic efficiency ($k_{cat}/K_{m,SAM}$) that is very similar to the *E. coli* enzyme. The PA-CFAS enzyme has a preference for acyl chain substrates linked to the PG backbone and was found to have higher activity on more highly unsaturated membranes. In contrast, catalytic activity and membrane binding by PA-CFAS do not appear to be strongly electrostatically driven under the conditions we tested, as the enzyme was found to exhibit similar reaction velocities over a wide range of ionic strengths (Fig. S8) and on both neutral (100% PE) and anionic (100% PG) membranes at pH 7.5 (Fig. 6*E*). Surprisingly, the electrostatic potential map of PA-CFAS calculated from the AlphaFold model suggests that the PA-CFAS enzyme is anionic in nature (Fig. S12). Thus, the preference for anionic PG substrate lipids and membrane surfaces (Fig. 6, *D* and *E*) observed in this study is curious. The *E. coli* enzyme has been shown to bind to vesicles with the aid of a positively charged Arg18/Lys48 dipeptide patch in the *N*-terminal lipid-binding domain (29). The PA enzyme appears to harbor a similar cluster of positively charged residues on the surface of the *N*-terminal domain (comprised of Arg17, Arg19, and Arg39), but the structure predicted for the *N*-terminal domain of the PA enzyme by AlphaFold is somewhat divergent from the structure observed for the EC enzyme. Moreover, the amino acid sequences of the lipid-binding domains among CFAS enzymes are not highly conserved (Fig. 7*B*), suggesting that this mode of membrane association may not be universal among CFAS enzymes. Nevertheless, the kinetic data reported here suggest that PA-CFAS is highly tolerant of various membrane compositions, a functional property that should enable PA to efficiently synthesize CFAs under a variety of different cellular growth and environmental conditions.

The exact structural mechanisms by which CFAS enzymes associate with membranes and extract unsaturated substrate acyl chains from the bilayer remain unknown. On the basis of structural and mutagenesis studies of the *E. coli* CFAS enzyme, Sauer et al. have proposed an “avidity” model for CFAS function where the two monomers of the EC-CFAS homodimer function asymmetrically (29). Namely, the *N*-terminal lipid-binding domain of one monomer anchors the dimer to the membrane, while the other monomer extracts a substrate acyl chain from the bilayer (*via* the lipid-binding domain) and catalyzes cyclopropanation (*via* the catalytic domain). The avidity model would also seemingly require structural plasticity in the two lipid-binding domains of the homodimer in order for the lipid-binding domain to carry out these two distinct functions (membrane anchoring and substrate extraction) presumably *via* distinct conformational states. Mutations to the disordered linker that separates the *N*-terminal and *C*-terminal domains also induce severe perturbations to EC-CFAS catalysis through an unknown mechanism. Interestingly, the only moderately conserved portion of the lipid-binding domain identified in our bioinformatic analysis (Fig. 7*B*) maps to the interface with the catalytic domain. These observations suggest that the CFAS dimer is likely a conformationally dynamic molecule and that proper dynamics and domain–domain interactions likely play a central role in CFAS function. Curiously, we identified CFAS homologs in our sequence similarity network that contain the lipid-binding domain but that lack the structural motifs needed for dimerization. If these enzymes are indeed lipid methyltransferases or CFA synthases, then they likely interact with bacterial membranes *via* a fundamentally different mechanism. Further investigation of the intriguing CFAS enzyme family will seek to clarify some of these unresolved structural and mechanistic issues.

## Experimental procedures

### Construction of *cfas* and oxyR deletion mutants

The unmarked deletion mutants for *cfas* and *oxyR* were made by the two-step allelic exchange method (60). For the *cfas* mutant, the upstream and downstream regions flanking the putative *cfas* (PA5546) ORF were amplified from the PAO1 gDNA using the primer pairs cfa-LA-out/cfa-LA-in and cfa-RA-in/cfa-RA-out, respectively (see Tables S3 and S4 for lists of all oligonucleotide sequences, strains, and plasmids used in this work). For the *oxyR* mutant, upstream and downstream regions flanking *oxyR* (PA5344) ORF were amplified using primers OxyR Outside B1, OxyR Outside B2, OxyR LA Inside Overlap, and OxyR RA Inside Overlap. For each gene deletion construct, the two amplicons of flvanking regions were fused using splice-by-overlapping extension PCR and then inserted into pEX18-Gm-GW (61) using Gateway cloning to form the respective suicide vectors containing the *cfas* or *oxyR* deletion alleles. The suicide vectors containing the *cfas* and *oxyR* deletion constructs were each introduced into the PAO1 parental cells by electroporation (for *cfas*) or biparental mating (for *oxyR*). Transformants were selected on LB agar with 50 μg/ml gentamicin, and merodiploid-resistant clones were subsequently counter-selected on LB agar (no salt) with 10 to 15% sucrose for *sacB*-mediated resolution of merodiploids. Gentamicin-sensitive sucrose-resistant clones were confirmed for the deletion of the *cfas* or *oxyR* genes by PCR and Sanger sequencing using primers cfa-conf-L1/cfa-conf-R1 and OxyR_Seq_F/OxyR_Seq_R, respectively.

### Cloning and expression of *cfas* promoter reporter

The promoter region (500 bp) of the *cfaS* gene (PA5546) was amplified from the PAO1 gDNA using the primers pCFA_Up_B1 and pCFA_Down_B5r. The *cfaS* promoter fragment and mCherry reporter gene (62) were cloned using Gateway recombinational cloning (63) into the final destination delivery vector pUC18-mini-Tn7T-Gm-GW, then chromosomally integrated as a single copy at the *attB* site (64) with the helper plasmid pTNS3 (65) into the various recipient *P. aeruginosa* strains. Transformants were selected with gentamicin 100 μg/ml and confirmed by PCR amplification of the insertion junction with primers PGLMS-DOWN and PTN7R. To measure *cfas* promoter activity, the strains harboring the integrated p_*cfas*_*-*mCherry reporters were grown in LB medium (37 °C, shaking 200 rpm), and absorbance (A_600_) and fluorescence (Ex. 587/Em. 620) were measured simultaneously using a multimodal plate reader (Tecan M200 Pro).

### *Cfas* promoter activity with paraquat challenge

The strains were first grown in 5 ml of LB medium for 18 h (37 °C, shaking 200 rpm), then diluted to A_600_ = 0.05 to inoculate 30 ml of LB in 250 ml flasks which were then incubated for 8 h (37 °C, shaking 200 rpm). At 8 h, the culture was split into two equal portions and was challenged either with a sublethal concentration of paraquat (1.25 mM final concentration) or left untreated as negative control. Flasks were returned to incubate (37 °C, shaking 200 rpm), and aliquots were sampled every 1 h to measure A_600_ and mCherry fluorescence (Tecan M200 Pro).

### Laurdan membrane fluidity assay

Laurdan generalized polarization assays were performed as done elsewhere (38) with minor modifications. Briefly, 5 ml of stationary phase cells were harvested from an 18 h overnight culture grown in 15 ml LB Miller medium (37 °C, shaking 250 rpm) by centrifugation at 7800*g* for 5 min and resuspended in 15 mM Tris–Cl pH 7.4 at an A_600_ = 0.4. After laurdan (0.75 μM final concentration) was added, samples were incubated in the dark (37 °C, shaking 250 rpm) for 1.5 h. Laurdan generalized polarization fluorescence (${GP}_{ex}$) was measured at Ex_350_/Em_435_ and Ex_350_/Em_500_ in a plate reader (Tecan M200 Pro) and calculated using the equation:

$${GP}_{ex}=\frac{\left(I_{435}-I_{500}\right)}{\left(I_{435}+I_{500}\right)}$$

Here, $I_{435}$ and $I_{500}$ represent the emission intensities at 435 nm and 500 nm, respectively. Background fluorescence from unbound dye was subtracted from the samples. The ${GP}_{ex}$ values reported in the text represent the mean ± SD calculated from four biological replicates pooled from two independent experiments.

### Cloning and expression of recombinant enzymes

The gene (PA5546), encoding the *P. aeruginosa* PAO1 CFAS enzyme (PA-CFAS), was amplified by PCR from the PAO1 genome using primers CFAHIS_F01 and CFAHIS_R01 (Table S3). Two additional PCR products were amplified from the *pET16b* vector using primer pairs *pET16bV2* and *pET16bR* (fragment 1) and *pET16bF* and *pET16bV1* (fragment 2). These three PCR products were then recombined to generate the PA-CFAS/pET16b construct using a 3-piece Gibson assembly (66). The assembled construct was confirmed by Sanger sequencing. Plasmids encoding the *E. coli* CFAS and LuxS enzymes (29) and the SAHNase enzyme were generous gifts from Prof. Robert Sauer (Massachusetts Institute of Technology) and Prof. Lynne Howell (University of Toronto), respectively. The plasmids were then transformed into either *E. coli* BL21(C43) (PA-CFAS, EC-CFAS, LuxS) or BL21(DE3) (SAHNase) for overexpression of the recombinant proteins as *C*-terminal fusions to a His_6x_-affinity tag. For each enzyme, an overnight culture was grown in LB supplemented with 100 μg/ml ampicillin for 18 h at 37 °C with shaking. This overnight culture was then used to inoculate fresh LB media (1:100 dilution) and the cultures were grown at 37 °C with shaking until the cells reached exponential phase (A_600_ = 0.7), at which point isopropyl IPTG was added to final concentrations of 0.5 mM (PA-CFAS, EC-CFAS, SAHNase) or 0.1 mM (LuxS) to induce protein expression. The cultures were then allowed to grow for an additional 4 h at 30 °C (PA-CFAS, EC-CFAS, SAHNase) or 18 h at 18 °C (LuxS). Cells were harvested by centrifugation at 5000*g* for 30 min at 4 °C and were stored at −80 °C for future purification.

### Purification of recombinant enzymes

For purification of recombinant PA-CFAS (Uniprot: Q9HT28), the frozen cell pellet was first resuspended in 100 ml of lysis buffer (50 mM Tris, pH 8.0, 100 mM NaCl, 10% glycerol, 20 mM imidazole, 0.1 mg/ml lysozyme) at 4 °C. The mixture was then sonicated on ice for 15 min (4.4 s pulse, 8.8 s between pulses) and was centrifuged at 15,000 rpm for 45 min to remove the cell debris from the supernatant. The supernatant was loaded onto a HisTrap FF column (Cytiva) pre-equilibrated with the lysis buffer and was washed with 10 column volumes of lysis buffer. The protein was eluted with a linear gradient of elution buffer (50 mM Tris, pH 8.0, 100 mM NaCl, 10% glycerol, 300 mM imidazole) from 0 to 100% over 60 min using a BioRad NGC Quest 10 Fast Protein Liquid Chromatography instrument with a flow rate of 1.5 ml/min. Fractions of 5 ml containing the protein were pooled together, concentrated, and further purified using size-exclusion chromatography with a HiLoad 16/600 Superdex 200 prep grade gel filtration column (Cytiva). Proteins were eluted isocratically in gel filtration buffer (50 mM Hepes, pH 7.5, 100 mM NaCl, 10% glycerol, 1 mM DTT) at a flow rate of 1 ml/min. The *E. coli* SAH nucleosidase enzyme (Uniprot: P0AF12) was expressed from the pPROEX HTa expression vector (67) and purified following the same protocol with the following modifications. The lysis buffer contained 50 mM sodium phosphate, pH 7.5, and 20 mM imidazole. The Ni-NTA elution buffer contained 50 mM sodium phosphate, pH 7.5, and 250 mM imidazole. The gel filtration buffer contained 50 mM Hepes, pH 7.5, 300 mM KCl, and 10% glycerol. The *E. coli* LuxS enzyme (Uniprot: P45578) was expressed from the pET21 expression vector (29) and purified following the same protocol as for the CFAS enzyme with the following modifications. The lysis buffer contained 20 mM Tris, pH 8.0, 500 mM NaCl, 5 mM imidazole. The Ni-NTA elution buffer contained 20 mM Tris, pH 8.0, 500 mM NaCl, 5 mM imidazole. The gel filtration buffer contained 20 mM Hepes, pH 7.5, 300 mM KCl, and 10% glycerol. Following gel filtration, all proteins were aliquoted, flash frozen with liquid nitrogen, and stored at −80 °C. The *E. coli* CFAS enzyme was expressed and purified as described by Sauer et al. (29). Protein concentrations were determined by measuring the A_280_ and using the calculated molar extinction coefficient.

### Verification of protein molecular weight using LC-ESI-MS

All samples were analyzed on a Waters Synapt G2-Si instrument. An aliquot of the frozen protein was thawed and diluted to 2 μM in solvent A (aqueous 0.1% formic acid). A 10 μl portion of sample was injected into an Acquity UPLC Protein C4 column (300 Å × 1.7 μM × 150 mm) pre-equilibrated in 5% solvent B (acetonitrile with 0.1% formic acid) for chromatographic separation. Samples were eluted with a linear gradient of 10 to 100% B over 20 min at a flow rate of 50 μl/min. The sample was then ionized by ESI using a capillary voltage of 3 kV, a sampling cone voltage of 40 V, a source offset voltage of 80 V, and a source temperature of 100 °C. The mass spectrometer was calibrated using 3 mg/ml NaI in 50:50 (v/v) water:isopropanol. Mass spectra over the range m/z = 100 to 2000 were collected in positive ion and sensitivity modes at a rate of 1 scan per second. [Glu-1]-fibrinopeptide B (GluFib) was used as an external standard. To determine protein molecular weights, mass spectra were deconvoluted using the MaxEnt1 function of MassLynx software (Waters) with the following parameters: resolution = 10.00 Da, uniform gaussian damage model width at half height = 1.00 Da, minimum intensity ratio = 33% for both left and right. Ions in multiple charge states were deconvoluted to their [M + 1]^1+^ charge state. The LC-MS data for SAHNase and LuxS are provided in Figure S13.

### Fatty acid extraction, derivatization, and GC-MS analysis

Planktonic cell cultures (5 ml) of wt PAO1 or the Δ*cfas* KO strain were grown overnight, centrifuged, concentrated in PBS, and lyophilized overnight. The dried biomass was then slowly dissolved in 2 ml of 20:1 (v/v) anhydrous methanol/acetyl chloride on ice followed by addition of 1 ml hexane. The mixture was then heated for 10 min at 100 °C, allowed to cool to room temperature, and the organic phase containing the fatty acid methyl esters (FAMEs) was separated in a 1:1 (v/v) H_2_O/hexane mix, dried under nitrogen stream, and stored at −20 °C until analysis. For GC-MS analysis, FAMEs were resuspended in 100 μl of hexane. A small volume of this sample (1 μl) was injected into a SCION single quad GC-MS instrument equipped with a HP-5MS column (30 m × 0.25 mm × 0.25 μm) with the following oven conditions: 80 °C for 1 min, 20 °C/min over 6.5 min followed by ramping at 10 °C/min to reach 300 °C. The transfer line temperature was 250 °C and the electron ionization source temperature was 200 °C. The MS data were collected in the range of 45 to 800 Da. Each FAME was identified by matching its electron ionization fragmentation pattern to the theoretical fragmentation spectrum available in the NIST database. Subsequently, the chromatographic peak areas of each FAME were determined and used to estimate the relative abundance of each species (Table S1).

### Preparation of *P. aeruginosa* phospholipid extracts

For lipidomic studies and *in vitro* PA-CFAS assays, we prepared PAO1 total lipid extracts as follows. A 5 ml overnight culture of PAO1 or Δ*cfas* was grown at 37 °C. On the following day, 2.5 ml of the overnight culture was used to inoculate a larger scale 250 ml culture. The culture was allowed to grow at 37 °C until the A_600_ reached the desired value (spanning the range 0.3–3.8), at which point 5 ml aliquots were removed. The cells in these aliquots were then harvested for 10 min at 5000*g* using a bench-top centrifuge. The pelleted cells were washed twice using 1 ml of Milli-Q water and membrane lipids were extracted following the Bligh-Dyer lipid extraction protocol (44) as follows: the washed lipid pellets were first resuspended in Milli-Q water to a total volume of 1 ml. Methanol (2.5 ml) was added to the cell suspension and the mixture was vortexed for 15 min before the addition of 1.25 ml of chloroform to obtain a final water/chloroform/methanol ratio of 0.8/1/2. After vortexing continuously for an additional 10 min, 1.0 ml of 1 M sodium chloride (NaCl) was added to the sample and vortexed for an additional 10 min. The mixture was centrifuged at 4000*g* for 2 min to allow better separation of the organic and aqueous layer. The organic (bottom) layer was collected. For better yield, the aqueous (top) layer was re-extracted with an additional 1.25 ml of chloroform. The organic layers were combined and dried under a nitrogen stream which resulted in a lipid film.

### LC-MS analysis of PA phospholipid extracts

Phospholipid extracts were analyzed by liquid chromatography electrospray ionization mass spectrometry. The lipid extract derived from 5 ml of PA cell culture was redissolved in 1 ml of LC-MS solvent A (60% acetonitrile, 40% 10 mM ammonium formate, 0.1% formic acid). A 20 μl portion of this sample was then injected into a Waters Acquity UPLC CSH C18 column (1.7 μM particle size, 1 × 100 mm column dimensions) and was resolved with the following gradient of solvent A and solvent B (88% isopropanol, 8% acetonitrile, 4% 10 mM ammonium formate, 0.1% formic acid) at 55 °C and a flow rate of 50 μl/min: 0 to 3 min, 0 to 62 %B; 3 to 8 min, 62 to 70 %B; 8 to 13 min, hold at 70 %B; 13 to 15 min, 70 to 89 %B; 15 to 21 min, hold at 89 %B; 21 to 22 min, 89 to 100 %B; 22 to 28, hold at 100 %B; 28 to 29 min, 100-0 %B; 29 to 35 min, 0 %B. The column effluent was directed to the ESI source of a Waters Synapt G2-Si mass spectrometer and was ionized in negative ion and resolution modes with capillary, cone, and source voltages of 2 kV, 30 V, and 50 V, respectively, at a 120 °C source temperature and 400 °C desolvation temperature. MS data were collected over an m/z range 100 to 2000 with a 0.5 s scan time. Following the acquisition of the MS data, target lipids were isolated using quadrupole selection and were fragmented by collision-induced dissociation in the trap region of the instrument by applying a linear collision energy ramp of 10 to 30 V over 0.5 s at an argon pressure of 0.0095 mbar. For semiquantitative analysis of PG and PE lipids, the extracted ion chromatogram peak areas for the PE and PG lipids of interest (Figs. S1 and S2) were extracted from the MS data and were analyzed with the QuanLynx module of MassLynx. All lipid quantities are reported as fractional abundances of the sum total of the extracted ion chromatogram peak areas for all of the lipids of interest. Validation experiments with PE and PG lipid standards established similar ionization efficiencies for both lipid classes under our MS conditions (Fig. S5). Thus, no further corrections to the signal intensities were made during the semiquantitative analysis.

### Native ion mobility mass spectrometry analysis of PA-CFAS

Prior to native ion mobility MS, the PA-CFAS enzyme was buffer exchanged from storage buffer into 200 mM ammonium acetate, pH 7.5, using a Microbiospin-6 column (Biorad). The sample (5 μM enzyme) was then manually loaded into a homemade nanospray emitter (with 1 μm tip opening) coated with 5 nm gold. The emitters were generated from thin-walled, 10 cm borosilicate capillaries (OD = 1 mm, ID = 0.78 mm, Harvard Apparatus) using a Sutter Instruments Model P-2000 tip puller with the following settings: heat = 350, filament = 4, velocity = 60, delay = 255, pull = 0. The emitter was then loaded into the Nanospray Source of a Waters Synapt G2-Si mass spectrometer, and the sample was ionized under gentle conditions: capillary voltage = 1.5 kV, cone voltage = 50 V, source offset = 50 V, source temperature = 50 °C. For ion mobility measurements, the trap DC bias was 45 V, and the IMS wave velocity and height were 650 m/s and 40 V, respectively. For collision-induced unfolding data, the trap collision energy was varied from 4 to 110 V using argon as the collision gas.

### Preparation of unilamellar vesicles for *in vitro* CFAS assays

All lipids (either purchased from Avanti polar lipids or extracted from PAO1) were first dissolved in chloroform. Lipids were mixed in the desired molar ratios and the chloroform was removed using a dry nitrogen stream. The remaining solvent was removed under vacuum for at least 2 h. The resulting lipid film was resuspended in buffer to a final concentration of 10 mg/ml. The lipid mixture was quickly frozen in lipid nitrogen and thawed in a water bath at room temperature for 5 min while sonicating. The freeze/thaw/sonication cycle was repeated five times to properly hydrate the lipids. Once the lipids were hydrated, the mixture was first extruded through a Whatman 400-nm polycarbonate membrane filter using an Avanti Extruder Set to remove large aggregates. This sample was then further extruded 21 times using a Whatman 100-nm polycarbonate membrane filter. The extruded unilamellar vesicles were prepared fresh and used in the same day for all CFAS biochemical assays.

### Coupled enzymatic assay for CFAS

For measuring the enzymatic activity of CFAS, we adapted the discontinuous colorimetric assay developed by Guianv’rc'h *et. al.* where the SAH product of the CFAS-catalyzed reaction is enzymatically converted to l-homocysteine (Hcy) *via* the combined action of SAH nucleosidase and LuxS (Fig. S6) (53). The Hcy is then reacted with Ellman's reagent (DTNB) to produce 2-nitro-5-thio-benzoate (TNB^2−^), which absorbs strongly at 412 nm. A standard assay mixture contained 1 mg/ml liposome, either 0.25 μM EC-CFAS or 2 μM PA-CFAS, 2 μM SAH nucleosidase, and 10 μM LuxS. The SAM, buffer, pH, and liposome compositions were altered for certain experiments as described in the text. Quench buffer (6 M urea, 0.5% v/v Triton-X 100) was prepared, aliquoted in 100 μl portions into the wells of a Costar clear 96-well plate, and was stored at 4 °C until the enzymatic reaction was initiated. The enzymatic reaction was initiated at 37 °C by adding either PA- or EC-CFAS. To measure initial velocities, 100 μl aliquots were removed at 2, 5, and 10 min and mixed thoroughly (1:1 v/v) with the quench buffer directly in the 96-well plate. The plate was transferred back to 4 °C between time points. After all time points had been quenched, 5 μl of a 4 mg/ml solution of DTNB was added to each well to give a final concentration of 336 μM. The plate was then covered in aluminum foil to prevent photodegradation of DTNB and was incubated for 10 min at room temperature. The absorption of the quenched solution was then measured at 412 nm and the concentration of TNB^2-^ anion was calculated using the reported extinction coefficient (14,150 M^−1^ cm^−1^). Control samples containing everything except the liposome were prepared and analyzed simultaneously with the assay samples. The absorption of the control sample was subtracted from the assay sample. The absorption readings were converted to concentration using a calibration curve prepared with l-cysteine (Fig. S14). All measurements were conducted in triplicate.

## Data availability

All data are contained within the manuscript. Any raw data reported in the manuscript are available upon request to the corresponding author (christopher.thibodeaux@mcgill.ca).

## Supporting Information

This article contains supporting information (22, 25, 27, 29, 61, 64, 65, 67, 68).

## Conflict of interest

The authors declare that they have no conflicts of interest with the contents of this article.
