# Supplementary material for: Defining the functional properties of cyclopropane fatty acid synthase from Pseudomonas aeruginosa PAO1
Source: J Biol Chem. 2024 Jul 31;300(9):107618. doi: 10.1016/j.jbc.2024.107618 (PMC11387697; doi:10.1016/j.jbc.2024.107618)
Supplement: Supporting Figures and Tables [file mmc1.docx]

**Supporting Information**

**Defining the Functional Properties of Cyclopropane Fatty Acid Synthase from *Pseudomonas aeruginosa* PAO1**

Vivian Ezeduru^1^, Annie R. Q. Shao^1^, Felipe A. Venegas^1^, Geoffrey McKay^2^, Jacquelyn Rich^2,3^, Dao Nguyen^2, 3, 4^, and Christopher J. Thibodeaux^1,5,*^

^1^Department of Chemistry, ^2^Research Institute of the McGill University Health Center, ^3^Department of Microbiology and Immunology, ^4^Department of Medicine, and ^5^Centre de Recherche en Biologie Structurale, McGill University

*To whom correspondence should be addressed

Christopher Thibodeaux

801 Sherbrooke St West

Montreal, QC, H3A 0B8

[christopher.thibodeaux@mcgill.ca](mailto:christopher.thibodeaux@mcgill.ca)

1-(514)-298-3637

**Supplemental Results**


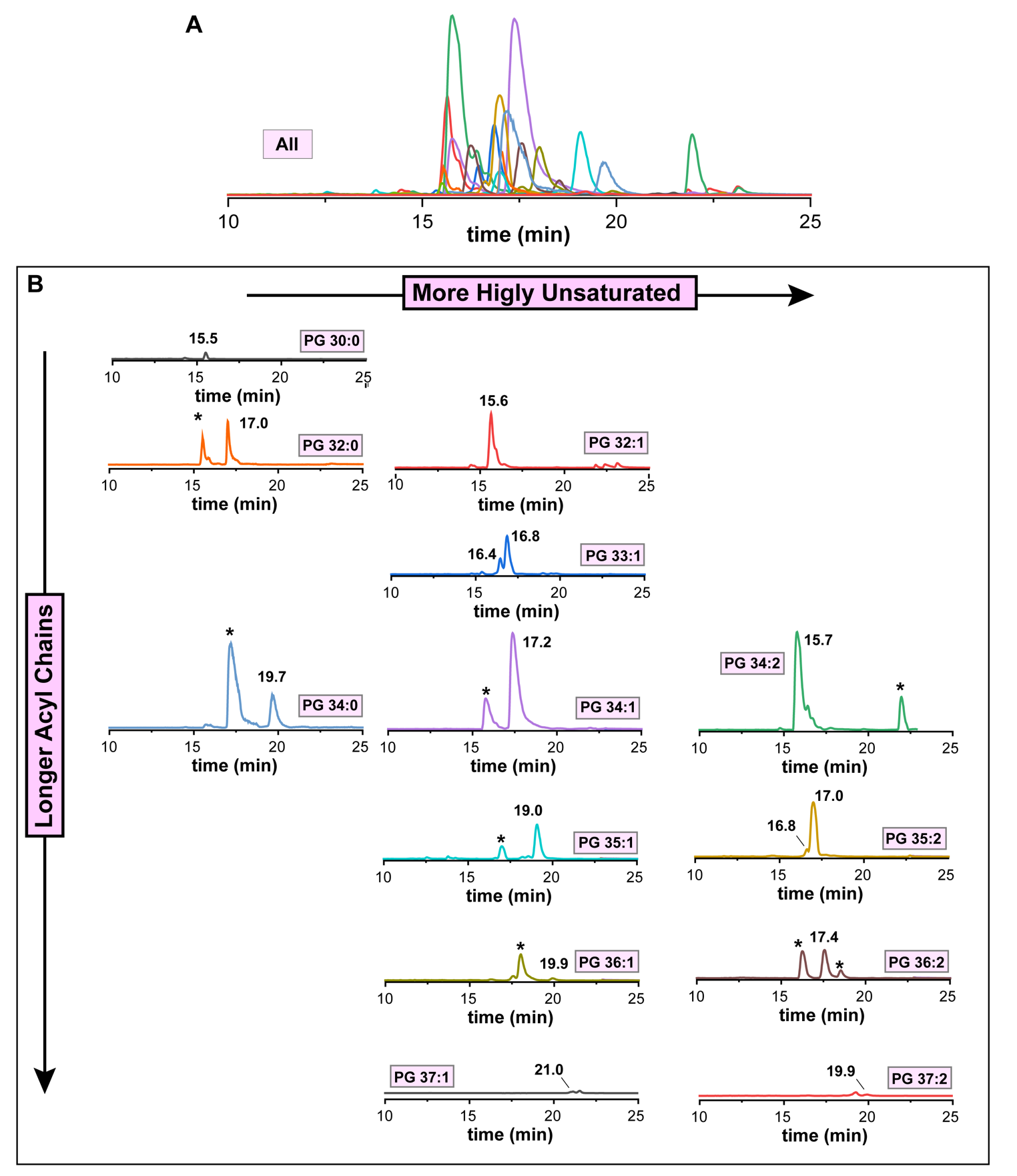


**Figure S1**. Extracted ion chromatograms (EICs) for phosphatidylglycerol (PG) lipids detected by LC-MS in PAO1 lipid extracts. The data shown are derived from a PAO1 cell culture harvested at an OD_600_ of 1.7. Extracts were resolved on a C18 column and were ionized in negative ion mode as described in the Materials and Methods. In panels (A) and (B), the EICs for all PG lipids are plotted on the same scale to better illustrate the relative abundance between compounds. Retention times for each compound are provided above the chromatographic peak. Each lipid was additionally fragmented by collision induced dissociation to identify the constituent acyl chains (Figure S3 and Table S2). As expected, retention times increased for lipids with longer, more saturated acyl chains. Asterisks mark signals for different compounds that coincidentally had the same m/z as the target ions.


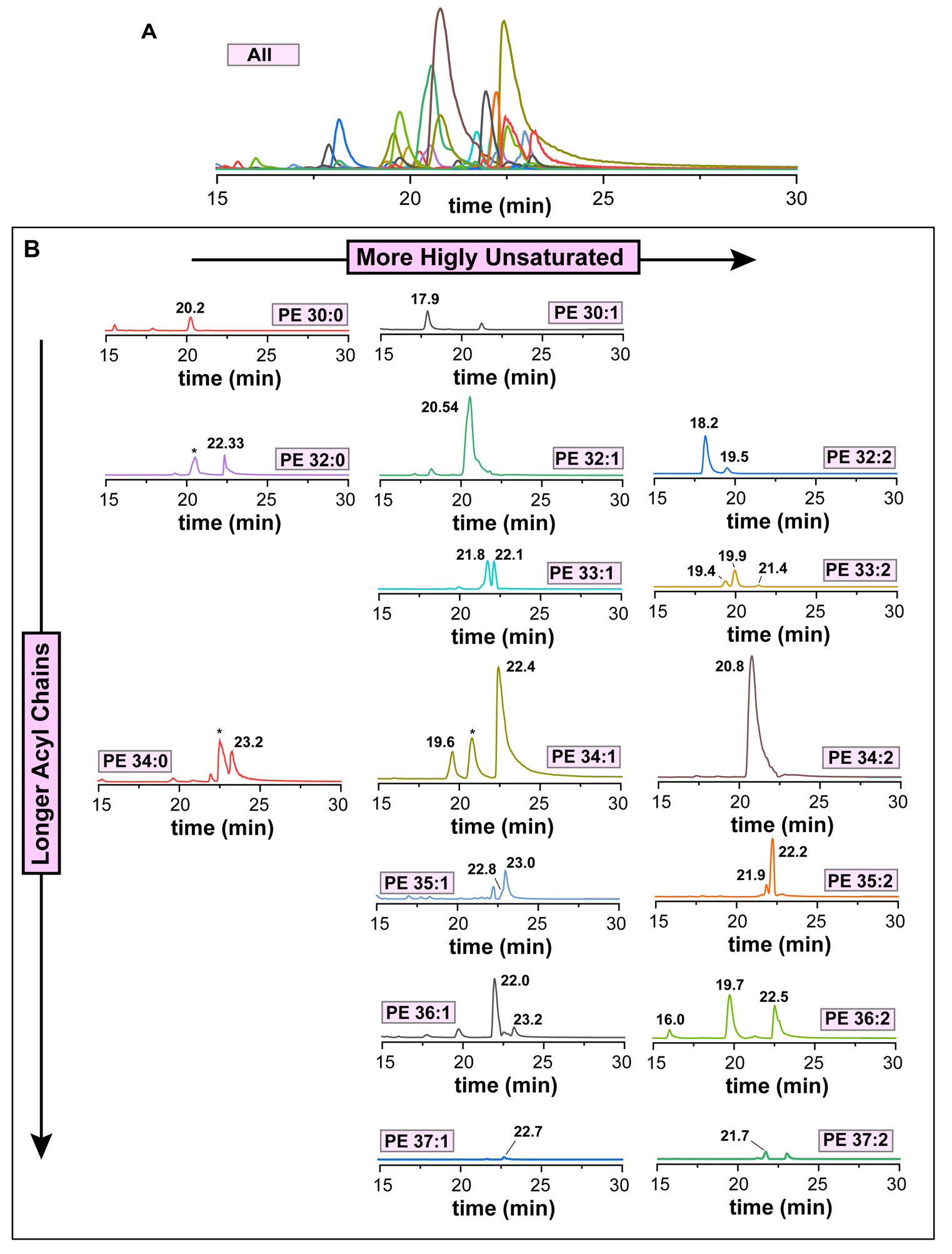


**Figure S2**. Extracted ion chromatograms (EICs) for phosphatidylethanolamine (PE) lipids detected by LC-MS in PAO1 lipid extracts. The data shown are derived from a PAO1 cell culture harvested at an OD_600_ of 1.7. Extracts were resolved on a C18 column and were ionized in negative ion mode as described in the manuscript. In panels (A) and (B), the EICs for all PE lipids are plotted on the same scale to better illustrate the relative abundance between compounds. Retention times for each compound are provided above the chromatographic peak. Each lipid was additionally fragmented by collision induced dissociation to identify the constituent acyl chains (Figure S4 and Table S2). As expected, retention times increased for lipids with longer, more saturated acyl chains. Asterisks mark signals for different compounds that coincidentally had the same m/z as the target ions.


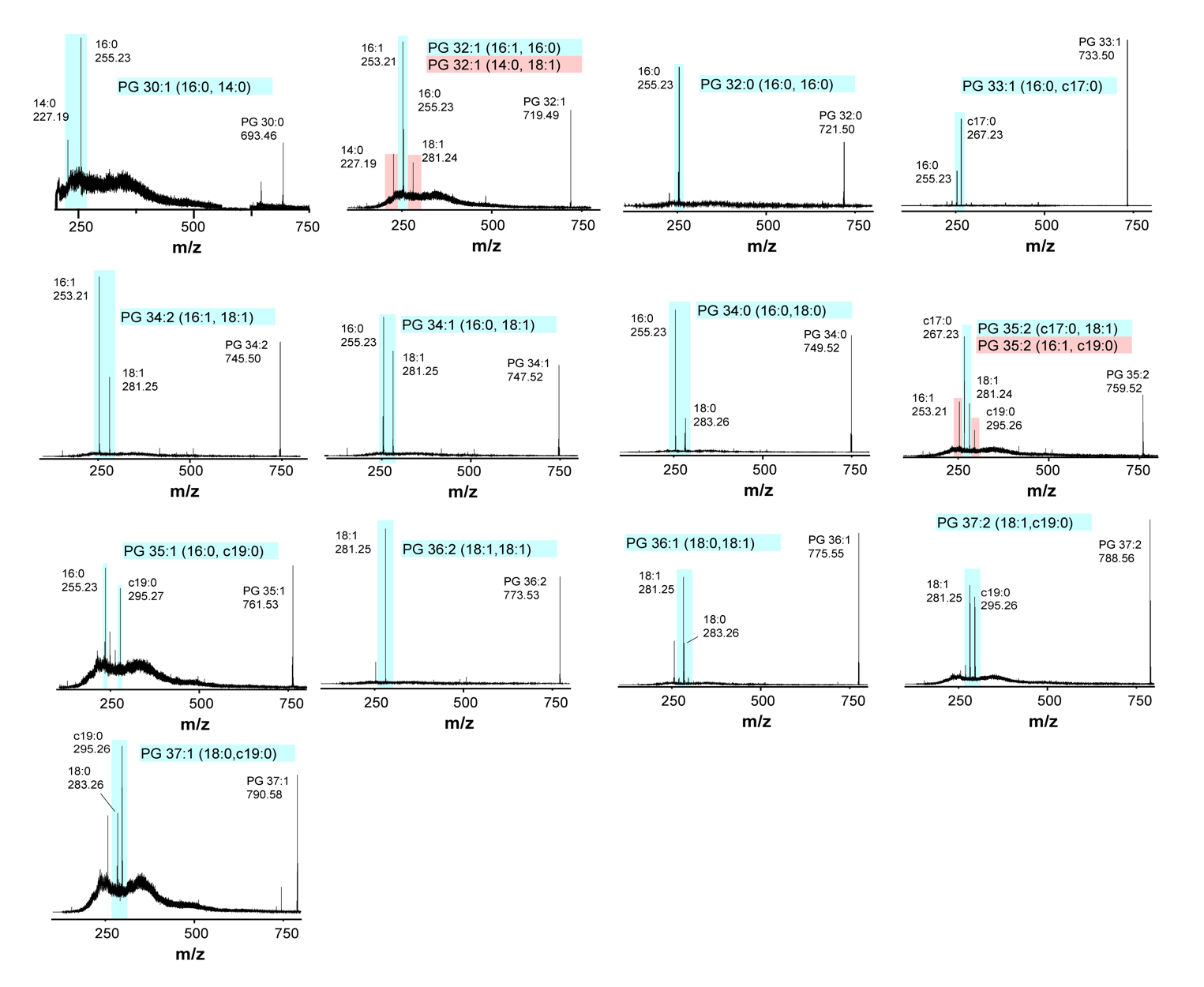
 **Figure S3**. Mass spectrometric fragmentation analysis of PG lipids. All fragmentation data are additionally summarized in Table S2. Each PG lipid was quadrupole selected and fragmented using argon as the collision gas. To achieve fragmentation, the collision energy was ramped from 10-30 V over a 0.5 s acquisition time. This collision energy ramp was chosen so as to maintain the intact unfragmented parent ion, which is indicated in each spectrum. The data shown correspond to the major chromatographic peaks in Figure S1. Minor isomers were also fragmented (summarized in Table S2). For each fragmented acyl chain, the detected m/z is provided along with the number of carbon atoms and double bonds. Acyl chains containing odd numbers of carbon atoms were not detected in the Δ*cfas* mutant by GC-MS analysis (Figure 2A). Thus, these acyl chains are tentatively assigned as cyclopropyl fatty acids. The attachment site of the two acyl chains on the glycerolphosphate backbone (*sn*-1 or *sn*-2) was not determined.


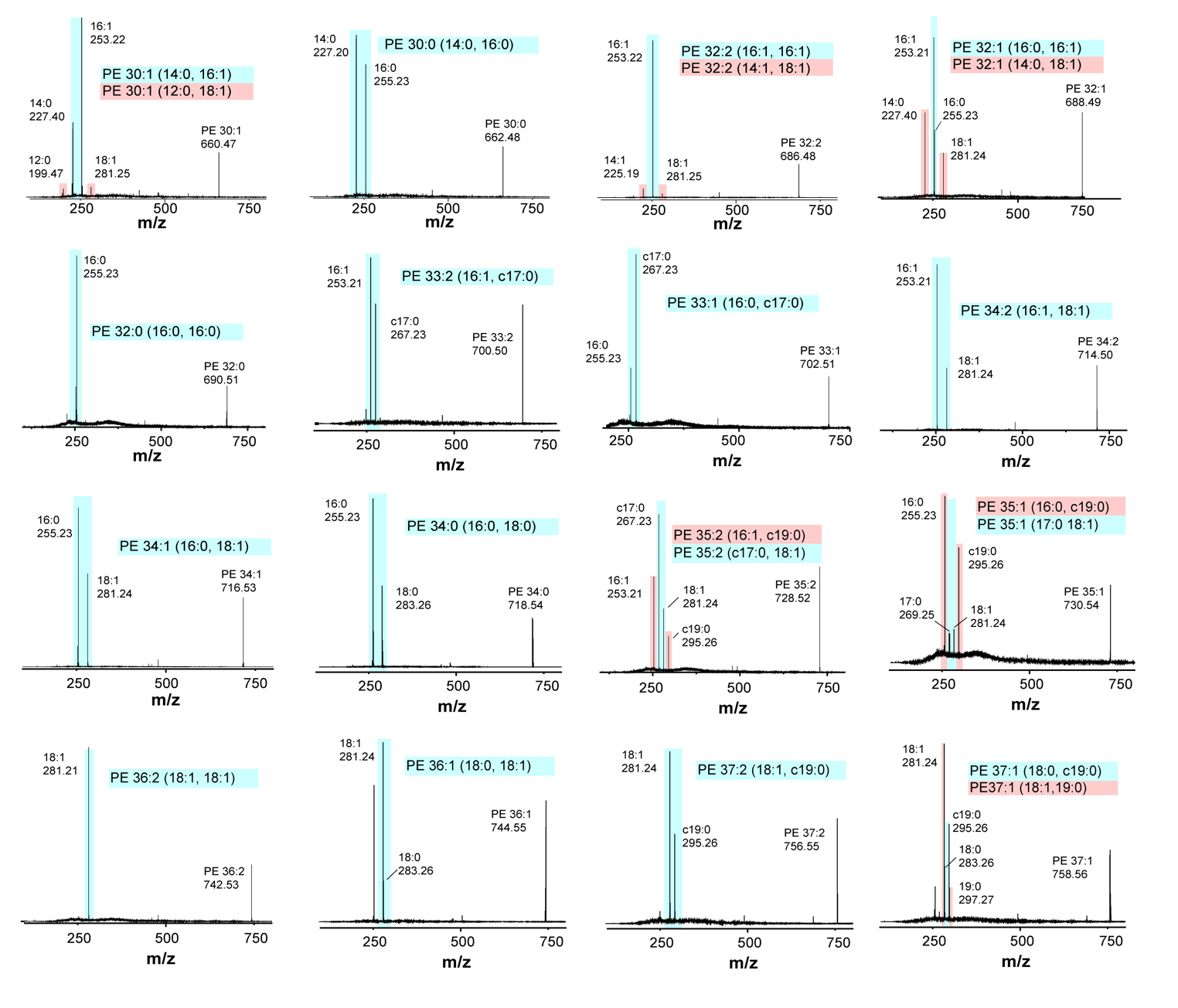


**Figure S4**. Mass spectrometric fragmentation analysis of PE lipids. All fragmentation data are additionally summarized in Table S2. Each PE lipid was quadrupole selected and fragmented using argon as the collision gas. To achieve fragmentation, the collision energy was ramped from 10-30 V over a 0.5 s acquisition time. This collision energy ramp was chosen so as to maintain the intact unfragmented parent ion, which is indicated in each spectrum. The data shown correspond to the major chromatographic peaks in Figure S2. Minor isomers were also fragmented (summarized in Table S2). For each fragmented acyl chain, the detected m/z is provided along with the number of carbon atoms and double bonds. Acyl chains containing odd numbers of carbon atoms were not detected in the Δ*cfas* mutant by GC-MS analysis (Figure 2A). Thus, these acyl chains are tentatively assigned as cyclopropyl fatty acids. The attachment site of the two acyl chains on the glycerolphosphate backbone (*sn*-1 or *sn*-2) was not determined.


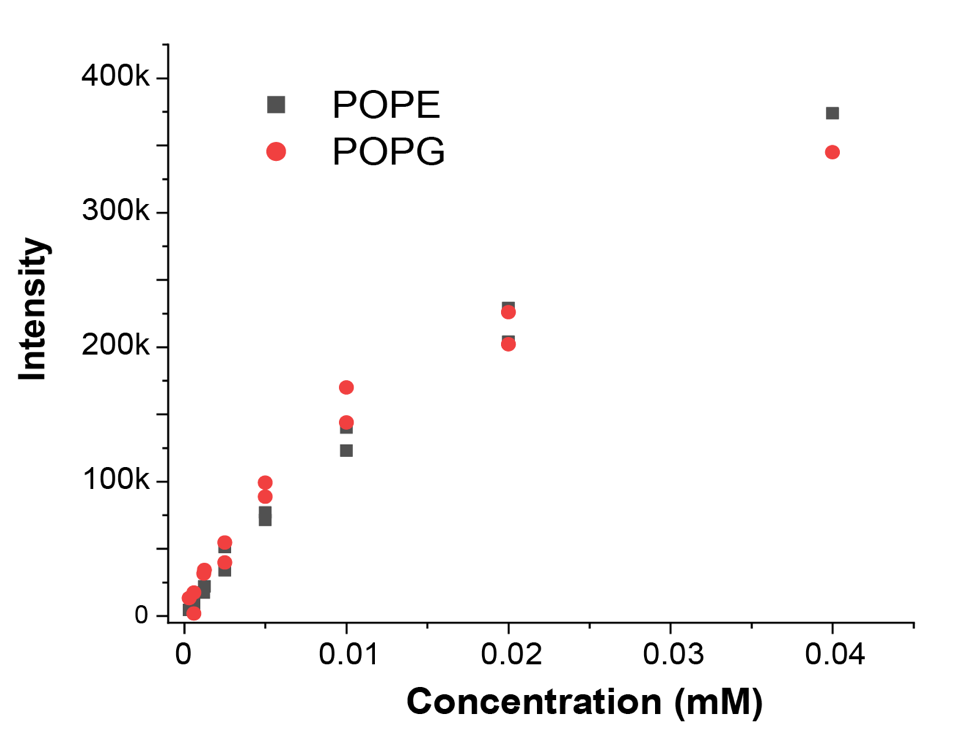


**Figure S5**. Establishing the relative ionization efficiencies of PE and PG lipids. A lipid standard containing 40 μM each of 1-palmitoyl-2-oleoyl-phosphatidylethanolamine (POPE) and 1-palmitoyl-2-oleoyl-phosphatidylglycerol (POPG) was prepared in duplicate and was then serially diluted. The standards were subjected to the same LC-MS conditions used for collecting the lipidomic data reported in Figure 4 of the Results and Discussion and the peak intensities of POPE and POPG were determined. The data show a very similar ionization response of both lipids under our LC-MS conditions. Thus, the fractional abundances determined from the extracted ion chromatograms in our semiquantitative analysis of the PAO1 lipid extracts are likely a good approximation of relative lipid concentrations.


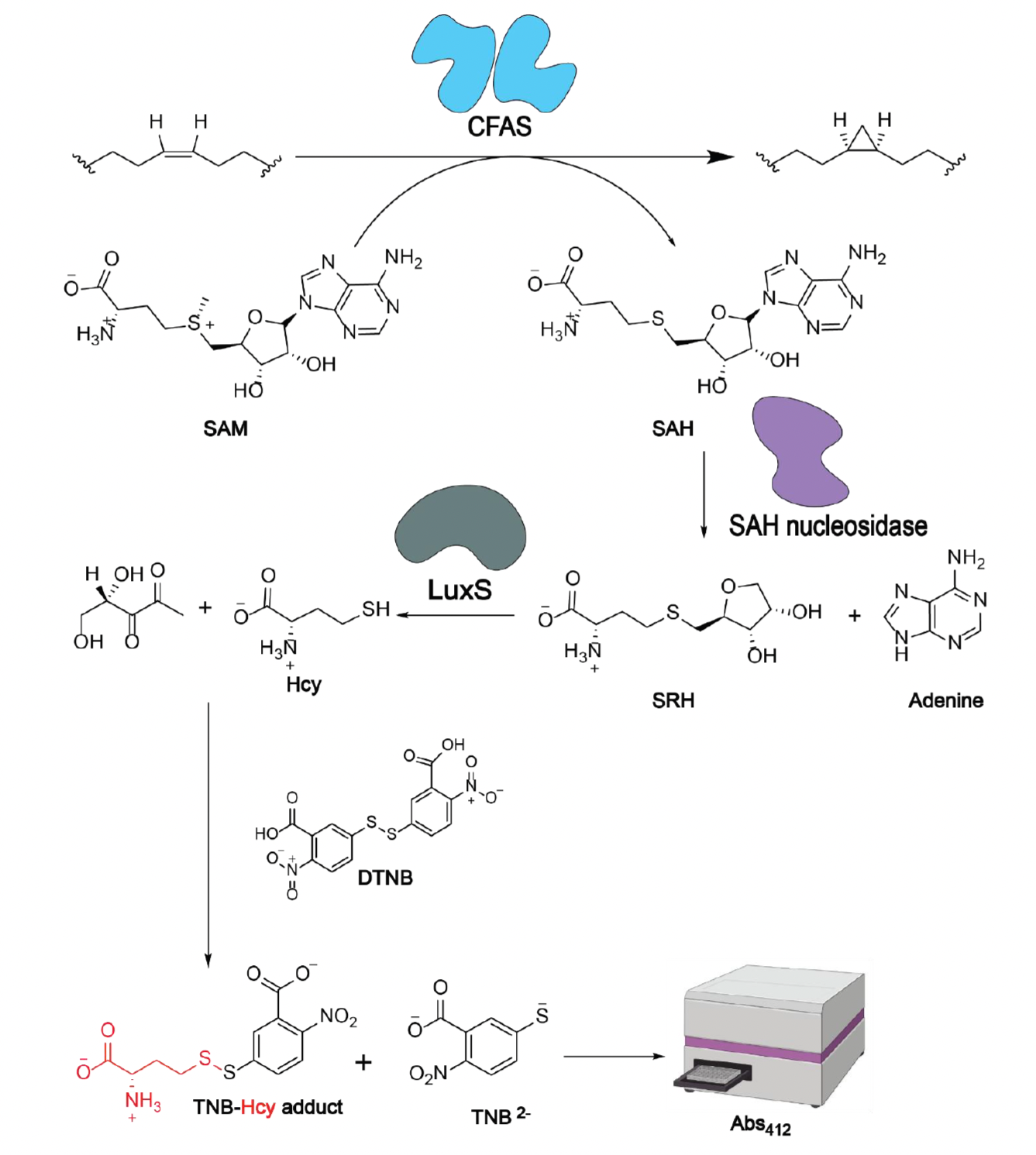


**Figure S6**. Schematic illustration of the colorimetric, coupled enzyme assay used to monitor CFAS activity.


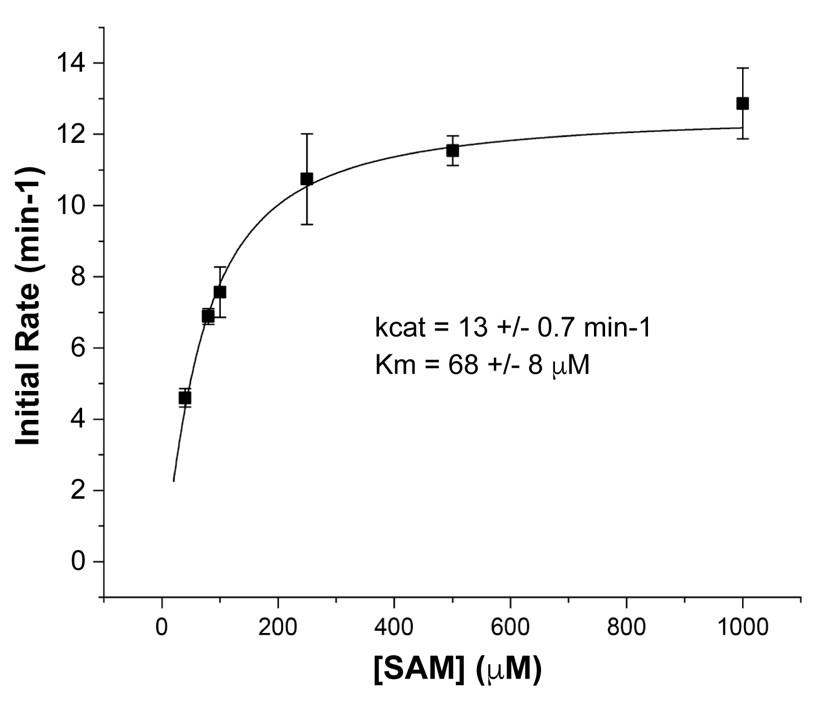


**Figure S7**. Steady state kinetic analysis of the *E. coli* CFAS enzyme. The reaction was performed with 0.25 μM EC-CFAS, 1 mg/mL DOPE:DPPG vesicles (60:40) in 50 mM HEPES, pH 7.5. The steady state kinetic parameters for EC-CFAS with SAM as the variable substrate have been previously determined at pH 7.5 by Booker and co-workers using 1-stearoyl-2-oleoyl-phosphatidylglycoerol (SOPG) as the substrate ($k_{cat}=7.3\pm0.3$ min^-1^, $K_{m}=90\pm13 \mu M$),(1) and by Ploux and co-workers using vesicles prepared from *E. coli* phospholipid extracts ($k_{cat}=2.5\pm0.07$ min^-1^, $K_{m}=70\pm2 \mu M$).(2)


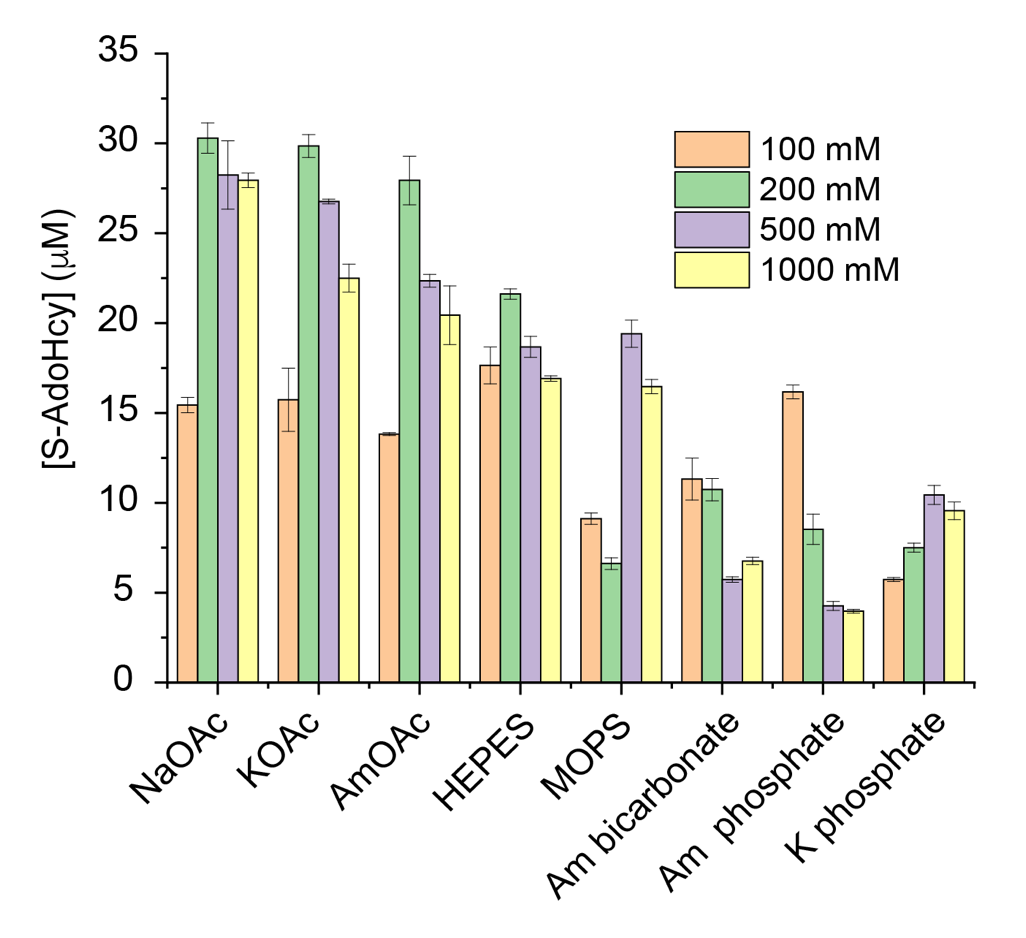


**Figure S8**. Screening for PA-CFAS activity in different buffers and ionic salts. Reactions contained 2 μM PA-CFAS, 1 mM SAM, 1 mg/mL vesicles composed of a 50:50 mixture of POPE and POPG, 2 μM SAHNase, and 10 μM LuxS in the indicated buffer/salt and the indicated concentration. Reactions were conducted for 20 min and were analyzed using the 96-well plate assay as described in the Methods.


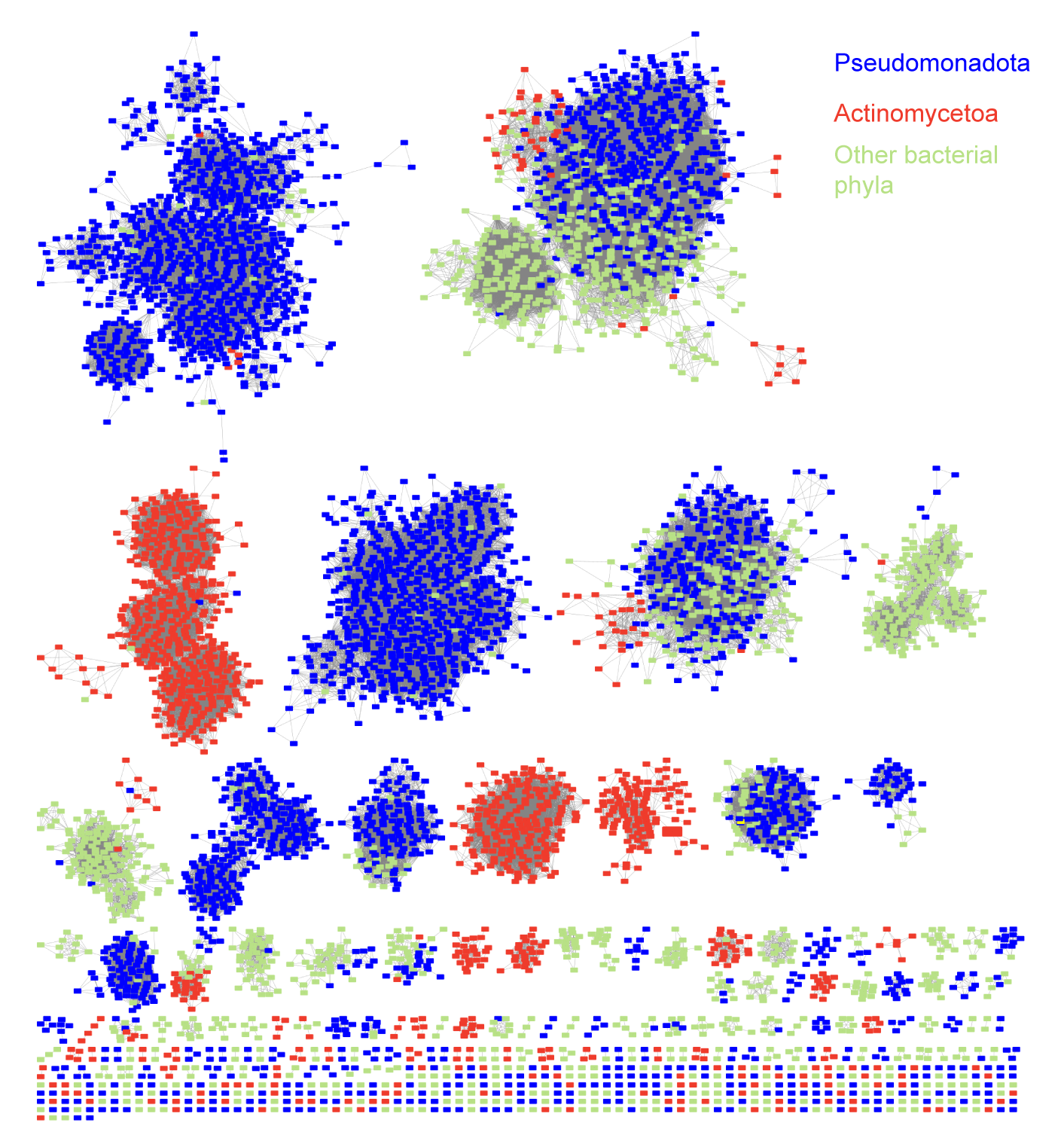


**Figure S9**. Distribution of cyclopropane fatty acid synthases across bacterial phyla. The large majority of enzymes come from the *Pseudomonadota* (57.5%, blue) and the *Actinomycetoa* (26.5%, red). The sequence similarity network was generated as described in the manuscript.


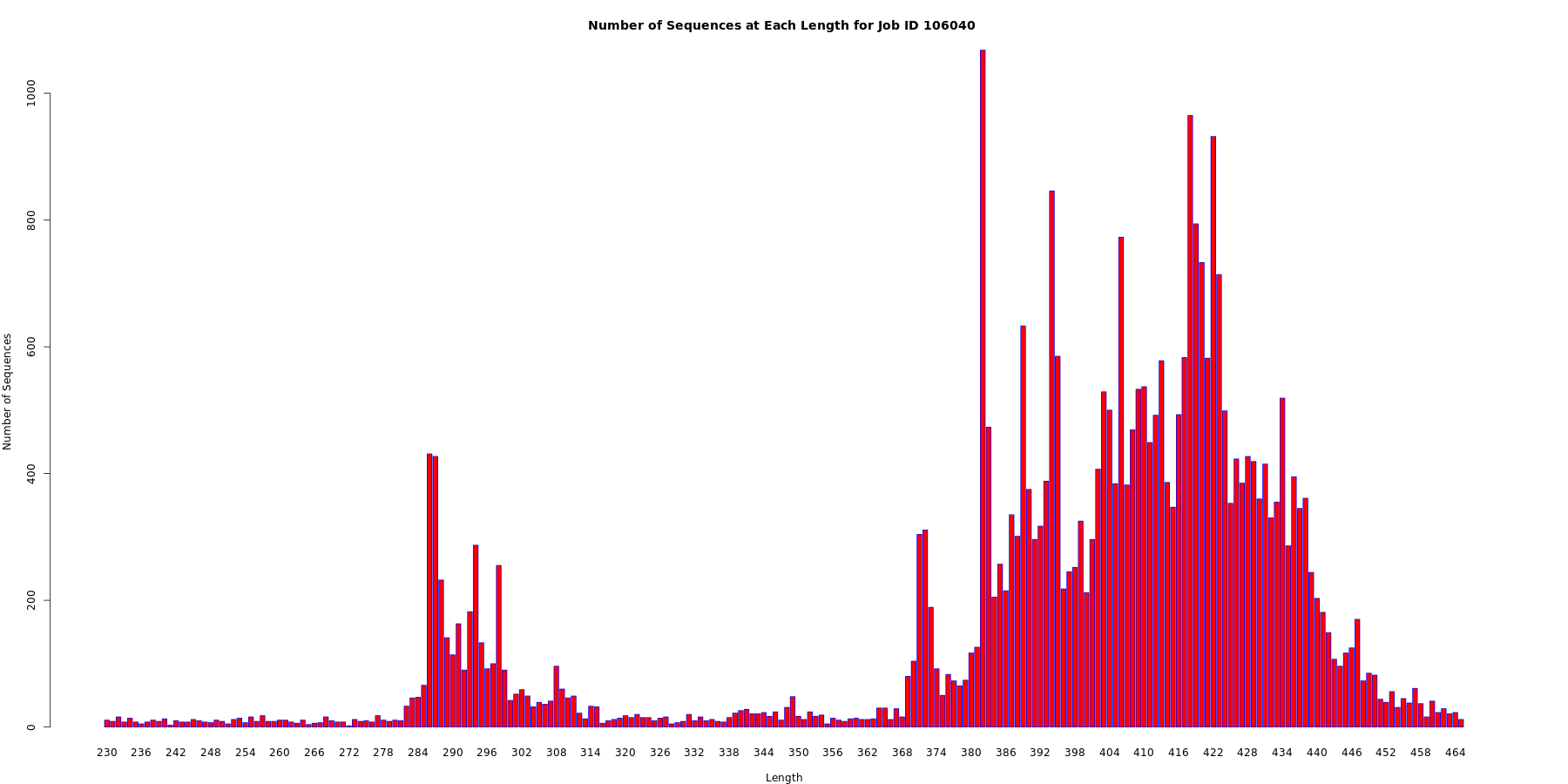


**Figure S10**. Amino acid sequence length distribution among enzymes containing the cyclopropane synthase domain (PF02353, IPR003333). The group of smaller enzymes do not form a specific clade and are distributed throughout the sequence similarity network (see Figure S11).


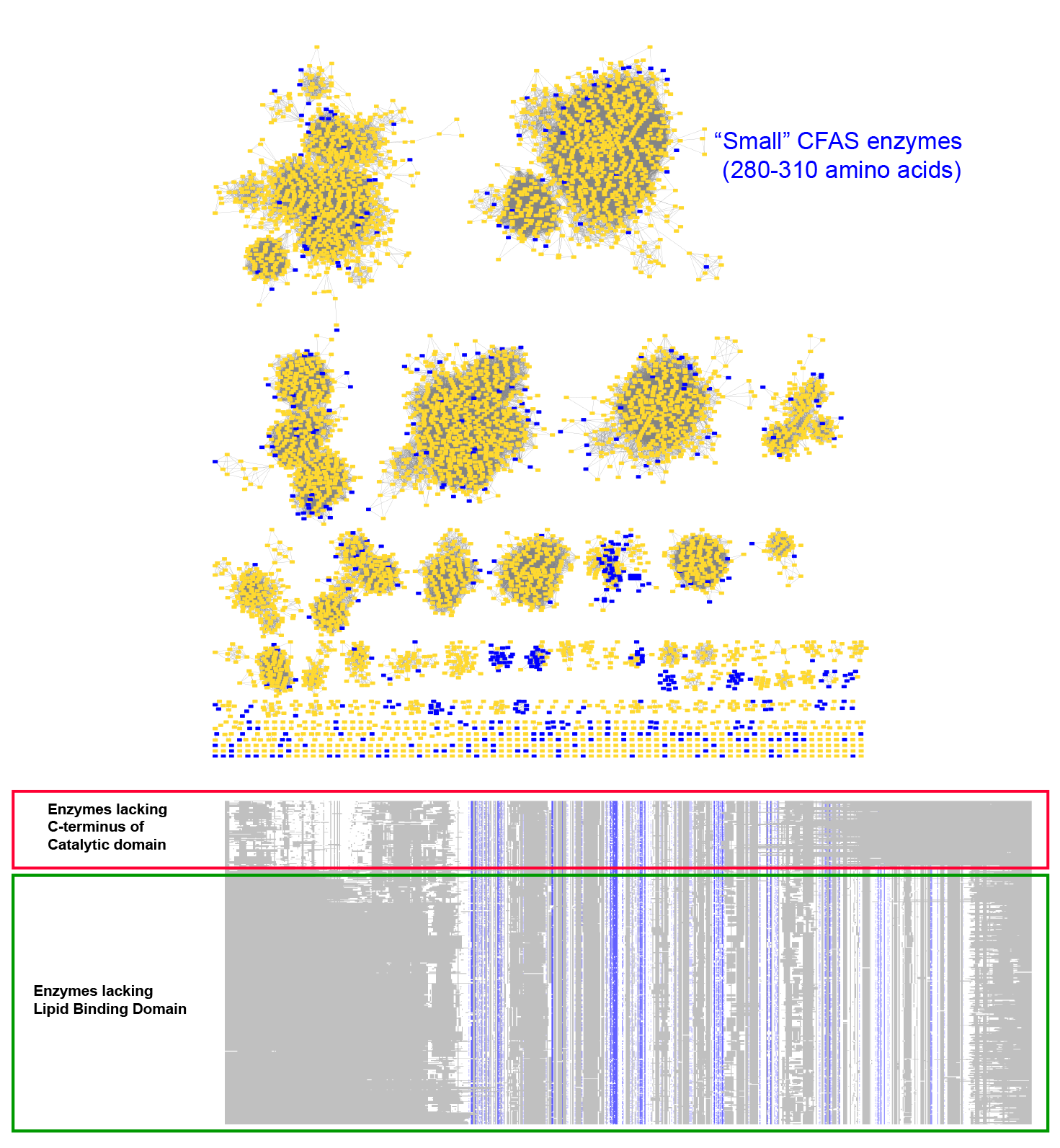


**Figure S11**. The CFAS sequences retrieved from the database fell into two distinct groups. The majority of CFAS enzymes (including *E. coli* CFAS and the PA-CFAS investigated in this work) are 350-500 amino acids in length and possess a bi-domain structure (yellow nodes, top). A smaller sub-population (about 10%) of proteins have sequence lengths of 280-320 (blue nodes). A sequence alignment of these “small” CFAS enzymes is shown at the bottom. The majority (80%) of these enzymes, such as the CmaA mycolic acid synthases from *M. tuberculosis* lack the *N*-terminal lipid binding domain (green box).(3) A smaller subset of enzymes (red box) contains an apparently intact lipid binding domain but lacks the *C*-terminus of the catalytic domain, which comprises the homodimerization interface and a significant portion of the interface between the lipid binding and catalytic domains observed in the EC-CFAS X-ray crystal structure.(4)


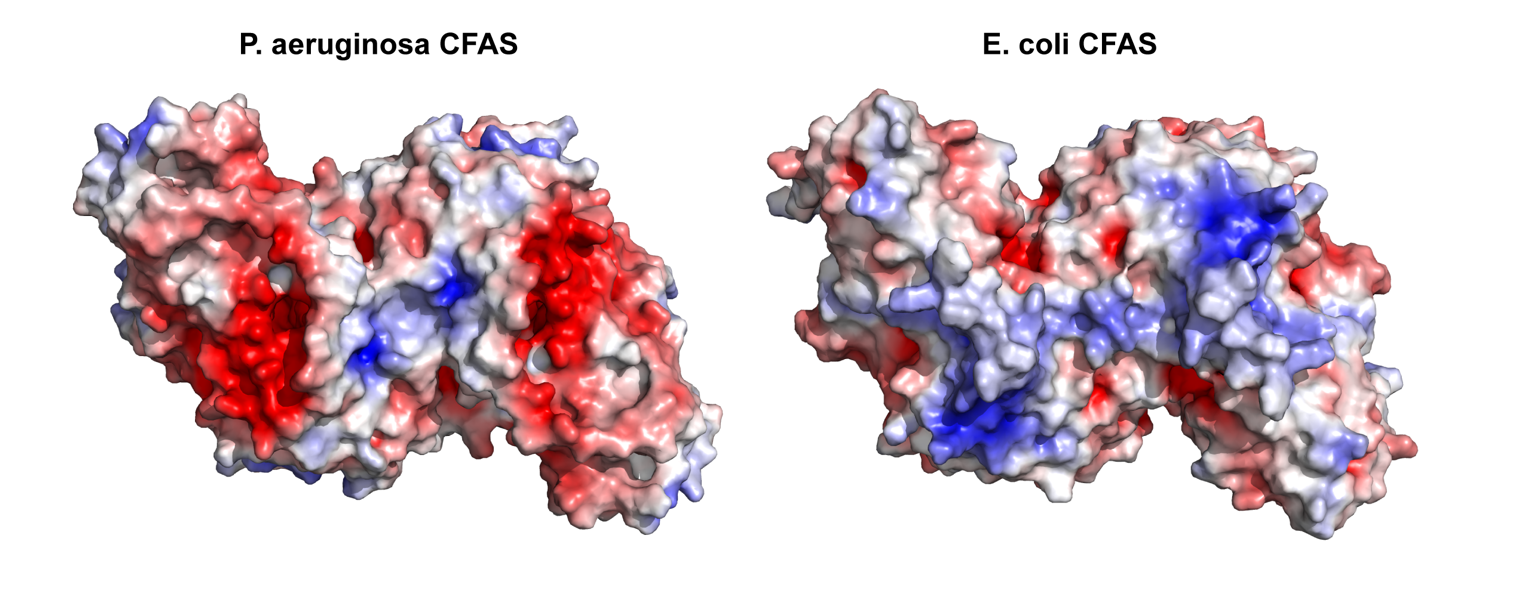


**Figure S12**. Electrostatic potential map calculated from the PA-CFAS AlphaFold model (left) and the EC-CFAS X-ray crystal structure (right). The putative membrane-binding surface is shown. Red and blue indicate negative and positive electrostatic potentials, respectively. Maps were calculated in PyMol using the default settings.


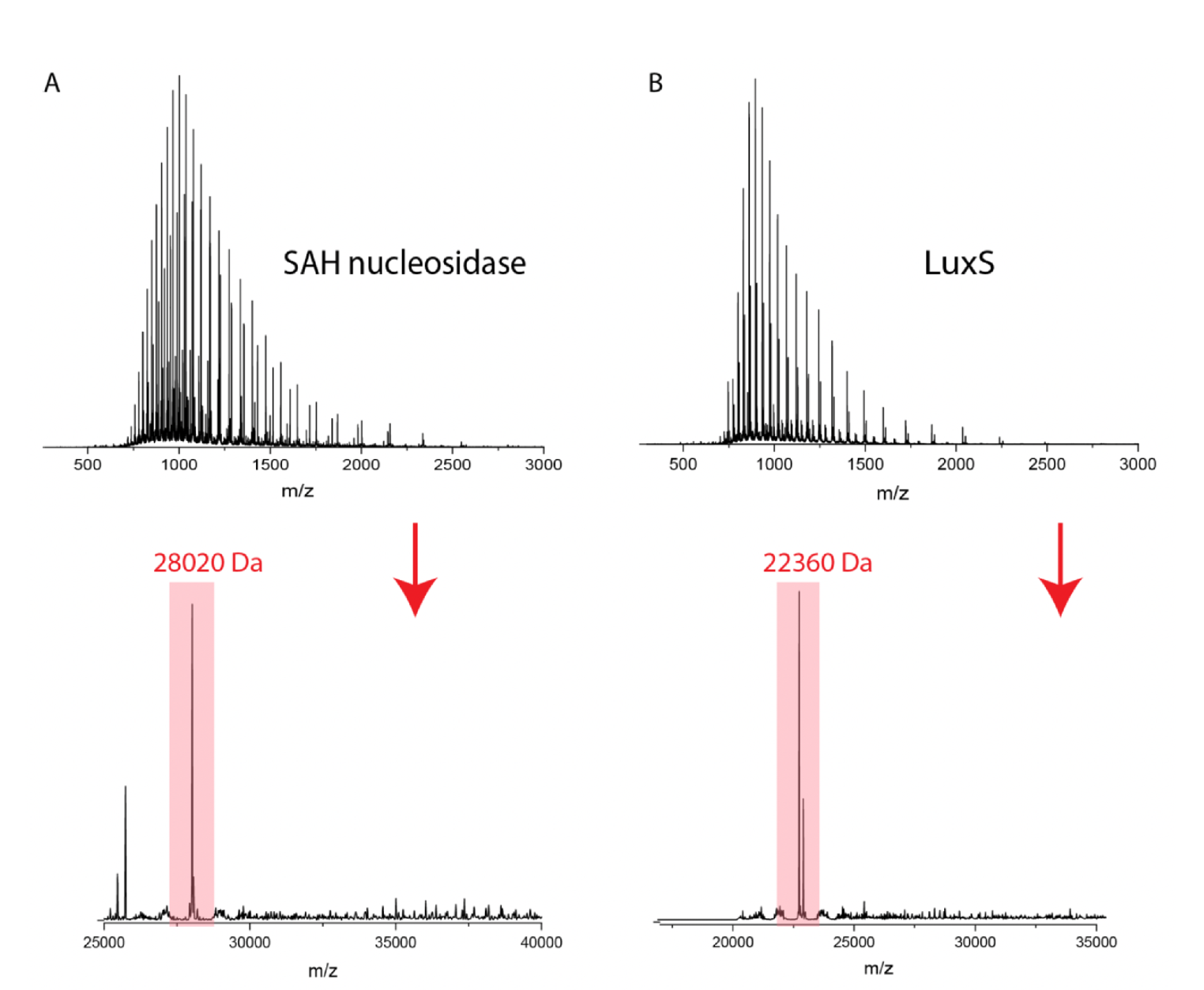


**Figure S13**. Molecular weight determination of SAH nucleosidase (panel A) and LuxS (panel B). Enzymes were expressed, purified, and analyzed by LC-ESI-MS as described in the Materials and Methods. The top panels show the raw ESI mass spectrum. The bottom panels show the charge state deconvoluted [M+H]^1+^ ions and indicate the observed average mass. The calculated average masses for SAH nucleosidase and LuxS are 28,025 Da and 22,360 Da, respectively.


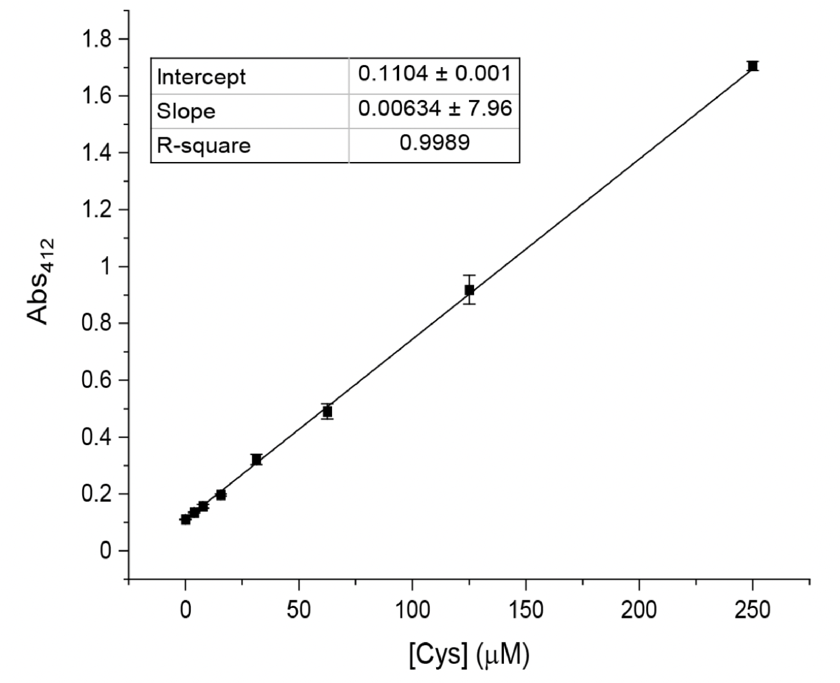


**Figure S14**. Calibration curve for thiol determination using the absorption of the 2-nitro-5-thio-benzotae dianion (TNB^2-^) at 412 nm. The TNB^2-^ anion is produced by reaction of Ellman’s reagent with thiols such as l-cysteine (used here to construct the calibration curve) or the homocysteine produced from the CFAS reaction product (*S*-adenosyl-l-homocysteine) by the combined action of SAH nucleosidase and LuxS. The Cys standards used for this calibration curve were prepared in CFAS assay buffer and were analyzed in the identical 96 well plate format used to analyze the CFAS assay samples.

**Table S1**. Quantification of fatty acid content in wt PAO1 and Δ*cfas* by GC-MS analysis. Fatty acid methyl ester (FAME) samples were prepared in triplicate from three separate stationary phase cultures of each strain and were analyzed and quantified by GC-MS as described in the manuscript.

|  | **wt PAO1** | **Δcfas** |
| --- | --- | --- |
| **Fatty Acid** | **Rel. Quantity (mean +/- S.E.)** | **Rel. Quantity (mean +/- S.E.)** |
| 16:1 | 5.7 $\pm$ 0.4% | 8.8 $\pm$ 0.4% |
| 16:0 | 38.5 $\pm$ 0.7% | 40.5 $\pm$ 1.0% |
| 18:1 | 44.3 $\pm$ 1.4% | 49.2 $\pm$ 0.4% |
| 18:0 | 0.8 $\pm$ 0.07% | 1.6 $\pm$ 0.5% |
| c17:0 | 1.5 $\pm$ 0.2% | N.D. |
| c19:0 | 9.2 $\pm$ 1.1% | N.D. |
| Total CFAs | 10.7% | - |
| Total SFAs | 39.3% | 42.1% |

**Table S2**. Summary of chromatographic, mass spectrometric, and tandem mass spectrometry fragment ion analysis of a lipid extract derived from a stationary phase PA01 cells harvested at an optical density (OD_600_) of 1.7. PE and PG lipids were quadrupole selected and fragmented by collision induced dissociation in negative ion mode to liberate the acyl chains from the glycerol backbone. The attachment site of the two acyl chains on the glycerolphosphate backbone (*sn*-1 or *sn*-2), the position of double bonds along the acyl chain, and the *cis*-/*trans*- stereochemistry of double bonds were not determined. These various possible constitutional and stereoisomers are likely responsible for the multiple chromatographic peaks observed for some of the phospholipids. Extracted ion chromatograms and fragmentation spectra for the PE and PG lipids are provided in Figures S1-S4. Further analysis of cardiolipins, alanyl-phosphatidylglycerol, and diphosphatidylglycerol lipids was not performed due to the low abundance of these species.

| **Phosphatidylethanolamine (PE) Lipids** | | | | |
| --- | --- | --- | --- | --- |
| **Lipid** | **Calc m/z** | **Obs m/z** | **Retention time (min)** | **Fragment Ions** |
| PE 30:1 | 660.46 | 660.47 | 17.89 | 14:0, 16:1 |
| PE 30:0 | 662.48 | 662.49 | 20.21 | 14:0, 16:0 |
| PE 32:2 | 686.48 | 686.48 | 18.19  19.51 (minor) | 16:1, 16:1  14:1, 18:1 |
| PE 32:1 | 688.49 | 688.51 | 20.54 | 16:0, 16:1  14:0, 18:1 |
| PE 32:0 | 690.51 | 690.51 | 22.33 | 16:0, 16:0 |
| PE 33:2 | 700.49 | 700.50 | 19.40 (minor)  19.92 | 15:1, 18:1  16:1, c17:0 |
| PE 33:1 | 702.51 | 702.52 | 21.75  22.13 | 15:0, 18:1  16:0, c17:0 |
| PE 34:2 | 714.51 | 714.50 | 20.75 | 16:1, 18:1 |
| PE 34:1 | 716.52 | 716.53 | 19.55 (minor)  22.41 | 16:0, 18:1  16:0, 18:1 |
| PE 34:0 | 718.54 | 718.55 | 23.18 | 16:0, 18:0 |
| PE 35:2 | 728.52 | 728.52 | 21.85  22.24 | 16:1, c19:0  c17:0, 18:1 |
| PE 35:1 | 730.54 | 730.54 | 22.99 | 16:0, c19:0 |
| PE 36:2 | 742.54 | 742.53 | 15.98 (minor)  19.71  22.53 (minor) | 18:1,18:1  18:1,18:1  18:1,18:1 |
| PE 36:1 | 744.55 | 744.55 | 21.95  23.16 (minor) | 18:0, 18:1  16:0, 20:1 |
| PE 37:2 | 756.55 | 756.57 | 21.67 | 18:1, c19:0 |
| PE 37:1 | 758.57 | 758.59 | 22.71 | 18:0, c19:0 |
|  | | | | |
| **Phosphatidylglycerol (PG) Lipids** | | | | |
| **Lipid** | **Calc m/z** | **Obs m/z** | **Retention time (min)** | **Fragment Ions** |
| PG 30:0 | 693.47 | 693.46 | 15.47 | 14:0, 16:0 |
| PG 32:1 | 719.49 | 719.49 | 15.57 | 14:0, 18:1  16:0, 16:1 |
| PG 32:0 | 721.50 | 721.50 | 17.04 | 16:0, 16:0 |
| PG 33:1 | 733.50 | 733.50 | 16.44 (minor)  16.77 | 15:0, 18:1  16:0, c17:0 |
| PG34:2 | 745.50 | 745.50 | 15.70 | 16:1, 18:1 |
| PG34:1 | 747.52 | 747.52 | 17.19 | 16:0, 18:1 |
| PG34:0 | 749.53 | 749.52 | 19.71 | 16:0, 18:0 |
| PG35:2 | 759.52 | 759.51 | 16.8 (minor)  17.0 | c17:0, 18:1  16:1, c19:0 |
| PG35:1 | 761.53 | 761.53 | 19.03 | 16:0, c19:0 |
| PG36:2 | 773.53 | 773.53 | 17.42 | 18:1, 18:1 |
| PG36:1 | 775.55 | 775.55 | 19.88 | 18:0, 18:1 |
| PG 37:2 | 788.56 | 788.56 | 19.90 | 18:1, c19:0 |
| PG 37:1 | 790.57 | 790.58 | 21.06 | 18:0, c19:0 |
|  | | | | |
| **Cardiolipins (CL)** | | | | |
| **Lipid** | **Calc m/z** | **Obs m/z** | **Retention time (min)** | **Fragment Ions** |
| CL 66:1 | 1377.98 | 1378.01 | 20.54 | Not analyzed |
| CL 68:4 | 1399.96 | 1400.01 | 20.45 | Not analyzed |
| CL 68:2 | 1404.00 | 1404.03 | 20.62 | Not analyzed |
| CL 68:1 | 1406.01 | 1406.04 | 21.68 | Not analyzed |
| CL 70:3 | 1430.01 | 1430.04 | 20.80 | Not analyzed |
| CL 70:2 | 1432.03 | 1432.06 | 20.79 | Not analyzed |
| CL 70:1 | 1434.04 | 1434.07 | 22.41 | Not analyzed |
| CL 72:3 | 1458.04 | 1458.08 | 22.22 | Not analyzed |
| CL 72:2 | 1460.06 | 1460.10 | 22.53 | Not analyzed |
| CL 74:2 | 1488.09 | 1488.14 | 22.15 | Not analyzed |
|  |  |  |  |  |
| **Alanyl-phosphatidylglycerol (aPG) Lipids** | | | | |
| **Lipid** | **Calc m/z** | **Obs m/z** | **Retention time (min)** | **Fragment Ions** |
| aPG 33:2 | 802.52 | 802.58 | 19.71 | Not analyzed |
| aPG 33:1 | 804.54 | 804.60 | 21.95 | Not analyzed |
| aPG 34:2 | 816.54 | 816.59 | 21.65 | Not analyzed |
| aPG 34:1 | 818.56 | 818.61 | 22.7 | Not analyzed |
| aPG 35:2 | 830.56 | 830.60 | 22.08 | Not analyzed |
| aPG 35:1 | 832.57 | 832.63 | 22.91 | Not analyzed |
| aPG 36:2 | 844.57 | 844.62 | 22.74 | Not analyzed |
|  |  |  |  |  |
| **Diphosphatidylglycerol (DPG) Lipids** | | | | |
| **Lipid** | **Calc m/z** | **Obs m/z** | **Retention time (min)** | **Fragment Ions** |
| DPG 32:1 | 799.45 | 799.49 | 15.33 | Not analyzed |
| DPG 33:1 | 813.47 | 813.51 | 15.90 | Not analyzed |
| DPG 33:0 | 815.48 | 815.53 | 17.62 | Not analyzed |
| DPG 34:1 | 827.48 | 827.52 | 16.91 | Not analyzed |
| DPG 34:0 | 829.50 | 829.57  829.53 | 16.48  18.83 | Not analyzed  Not analyzed |
| DPG 35:1 | 841.50 | 841.54 | 17.54 | Not analyzed |
| DPG 36:1 | 855.52 | 855.55 | 19.22 | Not analyzed |

**Table S3**. Oligonucleotide sequences used in this work (all sequences written 5'-3')

| **Item** | **sequence** |
| --- | --- |
| CFAHIS_F01 | GCAGCCGGATCCTCGAGCATTCAGGCGTACAGGTCGCGCCG |
| CFAHIS_R01 | CGGCCATATCGAAGGTCGTCATATGATTGCACAGCTCCCCTCTGAG |
| pET16bF | CTGCTGGCTACCCTGTGGAACA |
| pET16bR | GTGTTCCACAGGGTAGCCAGCAG |
| pET16bV1 | ATGCTCGAGGATCCGGCTGC |
| pET16bV2 | ATGACGACCTTCGATATGGCCG |
| cfa-LA-out | GGGGACAAGTTTGTACAAAAAAGCAGGCTACTTGCAGCGTAGCGTCGAGCTG |
| cfa-LA-in | TCTGAGTTGCTGTCCCTGCAACTGTACGCCTGAACGCCGGCG |
| cfa-RA-in | CAGTTGCAGGGACAGCAACTCAGAGGGGAGCTGTGCAATCATGCC |
| cfa-RA-out | GGGGACCACTTTGTACAAGAAAGCTGGGTAGTCACGGTGCTGCCGCTGC |
| pCFA_Up_B1 | GGGGACAAGTTTGTACAAAAAAGCAGGCTACCGCCCGCTCTTCCAGCACAT |
| pCFA_Down_B5r | GGGGACAACCTTTTGTATACAAAGTTGTGCCAAACCTCCTGGTCACAAGACG |
| cfa-conf-L1 | gcgatcaggtgcacgccga |
| cfa-conf-R1 | gcggatggccgccatgc |
| OxyR Outside B1 | GGGGACAAGTTTGTACAAAAAAGCAGGCTACAGGCCGTCGACGTAGGCGG |
| OxyR Outside B2 | GGGGACCACTTTGTACAAGAAAGCTGGGTAGCCTGTTGGCTCAACTGGCG |
| OxyR LA Inside Overlap | GTCATGCTATTTGCGGTTGTTCCTGGCGCAGTTCGGTGAGGGTCAT |
| OxyR RA Inside Overlap | ATGACCCTCACCGAACTGCGCCAGGAACAACCGCAAATAGCATGAC |
| OxyR_Seq_F | CCAGCCGCCATCGGTCTGC |
| OxyR_Seq_R | GGCCTGTTGGCTCAACTGGCG |

**Table S4. Strains and plasmids**

| **ID** | **Genotype or plasmid** | **description** | **Reference** |
| --- | --- | --- | --- |
| DN507 | DB3.1 pEX18-Gm-GW | *E. coli* containing Gateway compatible suicide plasmid for allelic exchange in *P. aeruginosa* | (5) |
| DN509 | DB3.1 pUC18-miniTn7T-Gm-GW | *E. coli* containing Gateway compatible miniTn7T plasmid for chromosomal integration in *P. aeruginosa* | (6) |
| DN1114 | DH5a pTNS3 | *E. coli* with helper plasmid | (7) |
| DN1253 | DH5α pUC18-mini-Tn7T2.1:: p_cfas_- mCherry | *E.coli* with miniTn7T2.1 plasmid containing p_cfas_- mCherry reporter | This study |
| DN1363 | DH5α pENTR::pEX18-Δ*oxyR* | *E.coli* with pEX18 suicide plasmid containing *oxyR* deletion construct | This study |
| DN1680 | DH5α pEX18Gm-GW -CFA-SOE | *E.coli* with pEX18 suicide plasmid containing *cfas* deletion construct | This study |
| DN276 | PAO1 (WT) | Wildtype PAO1 *P. aeruginosa* strain | (8) |
| DN1680 | Δ*cfas* | Unmarked in-frame *cfas* deletion mutant, parental strain PA01 | This study |
| DN1511 | Δ*oxyR* | Unmarked in-frame *oxyR* deletion mutant, parental strain PA01 | This study |
| DN1293 | PAO1:: pUC18-mini-Tn7T2.1:: p_cfas_- mCherry | PAO1 WT strain with chromosomally integrated p_cfas_- mCherry | This study |
| DN1825 | BL21 pET16b-CFA-HIS | *E. coli* with pET16b expression vector containing recombinant *P. aeruginosa* His-tagged *cfas* under lac promoter | This study |
|  | pET21 - His6-TEV-CFA | pET21 expression vector containing recombinant *E. coli* His-tagged *cfas* under lac promoter | (4) |
|  | pLuxS | pPROEX HTa expression vector containing recombinant *E. coli* His-tagged 5′-methylthioadenosine/*S*-­adenosylhomocysteine nucleosidase | (9) |

**References**

1. Iwig, D. F., Grippe, A. T., McIntyre, T. A., and Booker, S. J. (2004) Isotope and elemental effects indicate a rate-limiting methyl transfer as the initial step in the reaction catalyzed by Escherichia coli cyclopropane fatty acid synthase. Biochemistry **43**, 13510-13524

2. Courtois, F., and Ploux, O. (2005) Escherichia coli cyclopropane fatty acid synthase: is a bound bicarbonate ion the active-site base? Biochemistry **44**, 13583-13590

3. Huang, C. C., Smith, C. V., Glickman, M. S., Jacobs, W. R., Jr., and Sacchettini, J. C. (2002) Crystal structures of mycolic acid cyclopropane synthases from Mycobacterium tuberculosis. J. Biol. Chem. **277**, 11559-11569

4. Hari, S. B., Grant, R. A., and Sauer, R. T. (2018) Structural and Functional Analysis of E. coli Cyclopropane Fatty Acid Synthase. Structure **26**, 1251-1258

5. Choi, K. H., and Schweizer, H. P. (2005) An improved method for rapid generation of unmarked Pseudomonas aeruginosa deletion mutants. BMC Microbiol. **5**, 30

6. Choi, K. H., andSchweizer, H. P. (2006) mini-Tn7 insertion in bacteria with single attTn7 sites: example Pseudomonas aeruginosa. Nat.. Protoc **1**, 153-161

7. Choi, K. H., Mima, T., Casart, Y., Rholl, D., Kumar, A., Beacham, I. R. *et al.* (2008) Genetic tools for select-agent-compliant manipulation of Burkholderia pseudomallei. Appl. Environ. Microbiol. **74**, 1064-1075

8. Stover, C. K., Pham, X. Q., Erwin, A. L., Mizoguchi, S. D., Warrener, P., Hickey, M. J. *et al.* (2000) Complete genome sequence of Pseudomonas aeruginosa PAO1, an opportunistic pathogen. Nature **406**, 959-964

9. Lee, J. E., Cornell, K. A., Riscoe, M. K., and Howell, P. L. (2001) Expression, purification, crystallization and preliminary X-ray analysis of Escherichia coli 5'-methylthioadenosine/S-adenosylhomocysteine nucleosidase. Acta Crystallogr. D Biol. Crystallogr. **57**, 150-152
